# Surface Structure of Polar Silver Iodide (0001) in Various Liquids: No Indication for a Surface Reconstruction

**DOI:** 10.1002/cphc.202500904

**Published:** 2026-04-19

**Authors:** Kim Noelle Dreier, Annamaria Latus, Ralf Bechstein, Angelika Kühnle

**Affiliations:** ^1^ Physical Chemistry I, Faculty of Chemistry Bielefeld University Bielefeld Germany

**Keywords:** atomic force microscopy, ice nucleation, polar surface, silver iodide, surface reconstruction

## Abstract

Silver iodide (AgI) has long been known as a material that induces precipitation in clouds. The superior ice nucleation ability is commonly explained by the close lattice match between β‐AgI(0001) and the basal plane of ice. However, this surface is polar, indicating that a stabilization mechanism should be present. Here, we investigate the Ag‐terminated β‐AgI(0001) and the I‐terminated β‐AgI(000–1) surfaces in water and compare the results with images recorded in *n*‐dodecane, 0.1 M NaCl, and 0.1 M KI aqueous solutions. Strikingly, high‐resolution images consistently reveal a bulk‐truncated structure. These findings and observations at a larger scale indicate the absence of any periodic or triangular surface reconstruction. Although the observed structures reveal a variety of features and depend on the surface termination, some general conclusions can be drawn. First, in solvents with poor AgI solubility, only minor changes are observed. Second, the images taken in KI solution exhibit patterns that are characteristic of mineral dissolution and growth. Thus, even in a situation where AgI can and does dissolve, no indication for a surface reconstruction can be found. These results shed new light onto the surface structure of β‐AgI(0001), challenging the expectation that a surface reconstruction is present under ambient conditions.

## Graphical abstract

Silver iodide (AgI) is well known for its ice nucleating ability. The surface considered to be relevant in this respect is, however, polar. Polar surfaces are unstable and are anticipated to be stabilized, e.g., by surface reconstruction. Here, we show that no indication for a reconstruction can be found for AgI when the crystal is cleaved in various solvents. Our work challenges the expectation of a reconstruction to be present at ambient conditions.

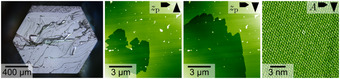

## 1 Introduction

Ice nucleation in clouds is decisive for the Earth's climate as it influences the Earth's radiation balance as well as precipitation formation [1]. While homogeneous ice nucleation requires temperatures as low as −38°C [2], heterogeneous ice nucleation is facilitated by the presence of ice nucleating particles (INP) and, thus, happens at significantly higher temperatures. Silver iodide (AgI) constitutes a prototypical material that has long been known for its excellent ability to induce ice formation at a temperature of about −4°C [3]. As early as 1947, B. Vonnegut identified silver iodide as an ice‐nucleating material from the close lattice match of the (0001) plane of β‐AgI to the basal plane of ice I_h_ [4]. In fact, silver iodide particles are extensively used for weather modification around the globe [5]. While attributing the ice nucleation ability to the close lattice match is tempting, it is well known that predicting ice nucleation capability based solely on a single descriptor can be misleading [6]. In case of β‐AgI (0001), the limited view on the lattice match might be even more puzzling, as the wurtzite (0001) and (000–1) surfaces belong to the Tasker III type, i.e., they are polar [7]. This is because, upon cleavage, the (0001) plane of the bulk‐truncated structure is entirely silver terminated, while the opposite (000–1) plane is iodine terminated, resulting in a macroscopic dipole moment. For these surfaces, it is generally accepted that a stabilization mechanism must be present, which alters the surface charge density and thereby compensates for the dipole moment [8]. Molecular dynamics (MD) simulations confirm the instability of the surface as they consistently report the structure to disintegrate when simulated in contact with water [9]. To circumvent this problem in the simulation, the surface atoms are often fixed at their bulk positions or constrained in place by an unrealistically steep harmonic potential [9, 10, 11, 12].

The polarization of a wurtzite crystal terminated by (0001) and (000–1) surfaces requires that the surface charge density in both surfaces is reduced by 25%. This can be achieved, for instance, by removal of one quarter of the terminating ions [8]. Well‐known stabilization mechanisms include periodic arrangements of vacancies [13], step edge formation leading to triangular reconstructions [14], adsorption of ions leading to nonstoichiometric surfaces [15], electronic mechanisms involving charge transfer between the surfaces [16], and disordered reconstruction patterns [17]. Indeed, for β‐AgI cleaved in ultra‐high vacuum (UHV), atomic force microscopy (AFM) images have recently revealed a surface reconstruction that depends on the termination of the surface [18]. For the Ag‐terminated (0001) surface, the removal of one quarter of all terminating Ag ions has been observed, while the reconstruction of the I‐terminated (000–1) plane is more complex. These findings suggest that the close lattice match with ice is maintained on the Ag‐terminated side, while it might be impaired on the I‐terminated side. Interestingly, the MD simulations with the atoms fixed in the bulk‐truncated positions have also indicated the Ag‐terminated side to be more active as compared to the I‐terminated side, although in this case, the two sides provide identical lattice match with ice I_h_ [9, 11, 12, 19]. Thus, further aspects beyond the lattice match need to be considered for understanding the ice nucleation ability of AgI. The influence of the charge on the ice nucleation has been addressed in literature with somewhat inconclusive results, with one MD study finding no influence upon varying the charge on the silver ions [11], while the other study reports the charge distribution to have significant impact on the ice nucleation [20]. Special active sites similar to those known for other ice nucleating agents such as feldspar [21, 22] have rarely been discussed for AgI so far. Defect sites such as step edges and pits have been shown to reduce the ice nucleation and growth rates in MD simulations rather than promoting them [23]. In a recent MD study, the effect of irregular surfaces resulting in spatially inhomogeneous electric fields has been investigated, indicating that metastable ice types might form as a precursor [24].

In clouds, the conditions differ from those in a UHV environment, prompting the question of whether the reconstruction mechanism observed in UHV is of significance in this context. Therefore, the investigation of the solid–liquid interface is of paramount importance as it is closer to the situation of ice nucleation in the immersion mode. While pure water has been found to be insufficient in an MD simulation for stabilizing the polar (0001) and (000–1) surfaces of β‐AgI, the presence of ions has been shown to be sufficient for polarity compensation [10]. Thus, the presence of ions in an aqueous environment might be an option for stabilizing the surface without the need of surface reconstructions. So far, our AFM images taken at the solid–water interface have, indeed, revealed an atomic structure compatible with the bulk‐truncated surface [25]. However, a faceted structure has been observed on a larger scale, which may potentially have resulted from a reconstruction mechanism. These previous AFM findings raise the question of whether a reconstruction (i) is absent in the presence of an aqueous environment, or (ii) is present but not discernible in AFM images captured at the solid–liquid interface. Furthermore, the absence of a reconstruction in the polar solvents water and ethanol [25] motivates experiments in a nonpolar solvent, as well as in electrolyte solutions to evaluate the significance of solvent polarity and ion activity. Another important aspect is AgI solubility, as one might argue that only solvents with high AgI solubility allow for reconstructions if these were thermodynamically favored.

Here, we present AFM images taken at the Ag‐terminated (0001) and the I‐terminated (000–1) surface after cleavage in various solvents. Besides pure water and a 0.1 M sodium chloride (NaCl) aqueous solution, both having a poor solubility for AgI, we investigate *n*‐dodecane as a solvent with even less AgI solubility. We compare the results obtained in these solvents with a sample cleaved and imaged in 0.1 M potassium iodide (KI) aqueous solution, featuring a high AgI solubility. High‐resolution images reveal atomically resolved images that are compatible with a bulk‐truncated structure, regardless of the solvent used. These atomic‐scale images are complemented by our images obtained at a larger scale, which do not show indications of a distinct surface reorganization. Although the images at larger scale exhibit a variety of different features and depend on the surface termination, several general statements can be made. In all used solvents, in which AgI is poorly soluble, only minor changes are observed. Those changes, which appear to be promoted by the scanning process, result in a surface with reduced density of step edges rather than evolving into a triangular reconstructed surface. For the KI solution, the step edges of the surface can be observed advancing and retreating in a manner characteristic of mineral dissolution and growth. Thus, AgI in water might be viewed as a frustrated sample, which is more or less unable to alter its surface structure, much alike the situation that is expected and observed for *n*‐dodecane. The situation is the same when adding NaCl, which does not change the solubility of AgI in the solution. For a KI solution, however, the situation is different. In this solvent, AgI is soluble due to complexation of Ag^+^ as a [AgI_2_]^−^ complex by excess iodide ions present in the solution. Interestingly, although the crystal can and does dissolve in this solvent, we observe no indications of surface reconstruction occurring, neither at the atomic scale nor at the micrometer scale. Instead, experiments in a 1 M KI aqueous solution show indications of the polar (0001) plane vanishing in favor of pyramidal shaped structures. While we cannot exclude the possibility that the reconstruction on the atomic scale is disordered and, hence, may be so subtle as to remain indiscernible in our AFM images, the consistent results observed for both cleavage planes across all investigated solvents do not provide compelling evidence for a periodic or a triangular reconstruction occurring. These results provide new insights into the surface structure of β‐AgI (0001), challenging the assumption that a surface reconstruction is necessarily present.

## 2 Methods

### 2.1 Growth and Preparation of Silver Iodide Single Crystals

**FIGURE 1:**
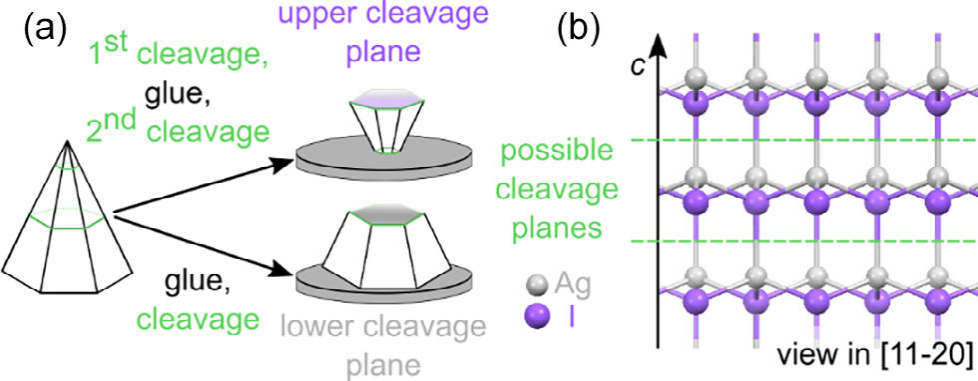
(a) Schematic illustration of the cleavage of single‐crystal AgI samples. (b) AgI crystal lattice viewed in [11, 12, 13, 14, 15, 16, 17, 18, 19, 20] direction. Cleavage at the indicated cleavage planes always results in a “lower”, Ag‐terminated (0001) plane and an “upper”, I‐terminated (000–1) plane.

Silver Iodide crystals were grown according to the method established by G. Cochrane [26] and D. R. Mills et al. [27]. A 3 M aqueous potassium iodide solution (>99%, CHEMSOLUTE a brand of Th. Geyer, Germany) was saturated with AgI (>99%, chemPUR, Germany) and filled into a long‐necked, cylindrical glass flask. After the solution had been overlaid with ultrapure water (Stakpure GmbH, Germany, 18.20 MΩcm), a glass rod was placed inside the flask and fixed in place with a rubber plug, which also sealed the flask. After leaving the solution in the dark for about 6 weeks, light yellowish, pyramidal shaped crystals with a hexagonal base plane were obtained. The crystals were rinsed with ultrapure water and stored in the dark. To prepare AgI samples for AFM experiments, AgI crystals were glued (UHU Super Glue Pipette, Germany) onto a magnetic sample holder and let dry at least overnight. Depending on which plane of the crystal was to be investigated, AgI samples were glued differently, as illustrated in Figure 1a. As confirmed by our X‐ray diffraction (XRD) measurements, which are presented in the Supplementary Materials of Ref. [18], the pyramidal AgI single crystals always have the same absolute orientation, in a manner similar to what is known for ZnO [28]. As shown in Figure 1a,b, cleavage parallel to the *c‐*axis of the crystal always results in a "lower" and an "upper" cleavage plane. For simplicity, in this work, these cleavage planes are thus referred to as the Ag‐terminated AgI(0001)‐Ag and the I‐terminated AgI(000–1)‐I plane, although the real surface termination in terms of chemical species cannot be determined from XRD and AFM experiments. Thus, to investigate the Ag‐terminated plane, crystals were glued with their hexagonal base plane onto the plate. To investigate the I‐terminated plane, the crystals had to be cleaved once with a razor blade, parallel to the base plane, and were glued with the resulting Ag‐terminated plane onto the plate. Before each experiment, glued samples were freshly cleaved with a razor blade inside the liquid. Apart from ultrapure water, *n*‐dodecane (99%, thermo scientific, Thermo Fisher Scientific, USA) and aqueous solutions of potassium iodide (≥99%, AppliChem GmbH, Germany), sodium chloride (≥99.5, VWR Chemicals, VWR International GmbH, Germany), and sodium iodide (≥99.5%, Sigma–Aldrich, Merck KGaA, Germany) were used. All chemicals were used as purchased.

### 2.2 Dynamic Atomic Force Microscopy Experiments

All AFM experiments were performed using a Cypher VRS1250 (Asylum Research, an Oxford Instruments Company, USA) and silicon cantilevers (TAP300GD‐G, Budget Sensors, Bulgaria) with a gold‐coated back side. After the AgI samples had been freshly cleaved in the respective solvent, the metal plate with the sample was transferred into the AFM liquid cell while taking care that the sample surface remains covered with solvent during the entire transfer. A liquid perfusion cantilever holder (Asylum Research, an Oxford Instruments Company, USA) was employed and further solvent was injected through the inlet tube using a glass syringe (Poulten & Graf, Germany). The outlet tube was sealed by a plastic syringe. To avoid photolytic degradation of the AgI samples during the AFM experiments [25, 29, 30], all light sources, except for the detection laser, were switched off as soon as possible. To achieve high‐resolution images on the atomic scale, AFM experiments were performed in the frequency modulation (FM) mode using a blue excitation laser for photothermal cantilever excitation, or in the amplitude modulation (AM) mode with piezo excitation. While the use of the blue laser shortened the measurement time due to photolytic degradation, the results at the atomic scale were the same as those obtained when using piezo excitation. All AFM experiments conducted on the micrometer scale were exclusively performed in amplitude‐modulation mode. For the large‐scale images, we refrained from using photothermal excitation [31] of the cantilever but used piezoelectric excitation instead. This approach is required to minimize the photolytic decomposition of the sample caused by the excitation laser. Although no measurable influence of the excitation laser was detected at the atomic scale, large‐scale images clearly demonstrated significant surface rearrangements upon irradiation with the blue laser. In fact, the facetted surface structure reported in our previous work [25] was observed only under irradiation with the blue laser. Therefore, we now ascribe the facetted structure to changes induced by the excitation laser. For image presentation, the large‐scale AM topography (*z*_p_ channel) images were leveled by a mean plane subtraction and fitting a plane through three points. Rows were aligned by a median of differences. Color bars are included in the images and, where appropriate, selected height profiles are provided in the Supporting Information. Some atomic‐scale images were corrected for thermal drift using the software *un*Drift [32] as indicated in the respective figure caption.

## 3 Results and Discussion

### 3.1 Atomic‐Resolution Imaging in Various Liquids

**FIGURE 2:**
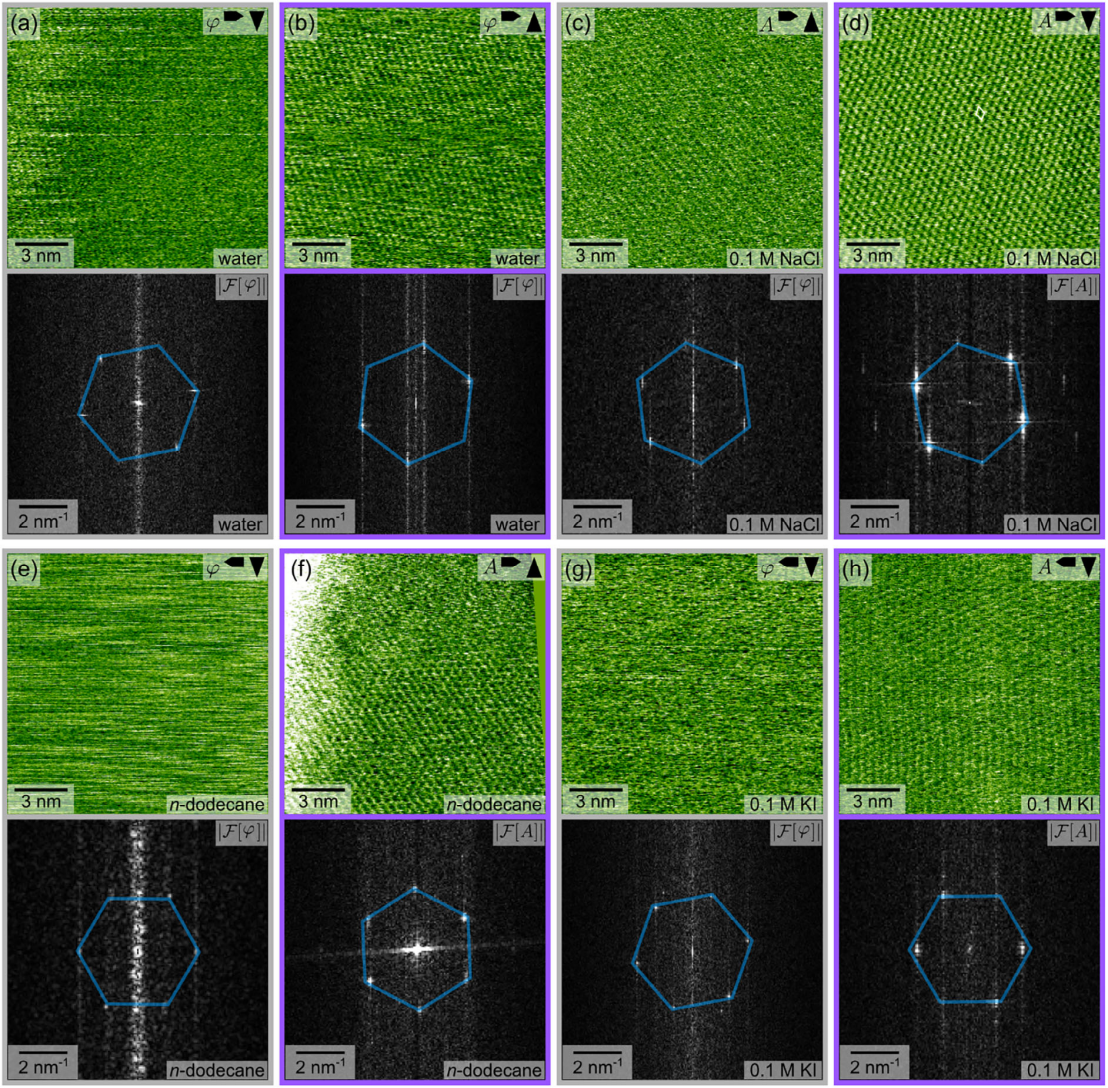
High‐resolution AFM images (phase shift *φ* or amplitude A) with the corresponding FFTs (F[*φ*] or F[*A*]) of the (0001)‐Ag plane (gray frames) and the (000–1)‐I plane (purple frames). The images were recorded in (a,b) water, (c,d) 0.1 M NaCl aqueous solution, (e,f) *n*‐dodecane, and (g,h) 0.1 M KI aqueous solution, respectively. Except for (e), all AFM images were corrected for linear drift using *un*Drift [32]. In (d), the corresponding unit cell is marked by a white rhombus. The blue hexagon in the FFTs was created based on the bulk unit cell parameters obtained from XRD measurements [18] and indicates the positions of spots that correspond to the reciprocal bulk unit cell of β‐AgI. As can be seen from the FFTs, the spots obtained from the AFM images excellently match with the XRD values for the bulk unit cell. The arrows in the upper right corner of the AFM images indicate the fast and slow scan direction.

To investigate the atomic surface structure of AgI when kept in various solvents, we cleaved and imaged our samples in pure water, 0.1 M NaCl aqueous solution, and *n*‐dodecane as well as in 0.1 M KI aqueous solution, respectively. We made an effort to ensure that the sample remained in the corresponding solvent after cleavage and never came into contact with air. Figure 2 presents AFM images along with their corresponding fast Fourier transforms (FFTs) obtained from the Ag‐terminated (0001) and I‐terminated (000–1) cleavage planes kept in the given solvent. The images taken on the Ag‐terminated side are marked with a gray frame, while the images taken on the I‐terminated side are marked with a purple frame. The respective solvent is indicated in the AFM image. As can be seen, our high‐resolution images reveal an atomic structure on both cleavage planes in all solvents. To determine whether the observed structure corresponds to the bulk‐truncated lattice, we compare the spot positions obtained in the FFTs with the expected spot positions for the bulk‐truncated crystal. To this end, we added a blue hexagon in the FFT frames, which is derived from the bulk lattice as obtained from the XRD data [18]. As can be seen, the positions of the spots obtained from the AFM images are in excellent agreement with the bulk unit cell. The bulk‐truncated lattice parameters are revealed in all solvents used, irrespective of whether the experiments were conducted in FM mode with a blue excitation laser or in AM mode using piezoelectric excitation.

As a reconstruction has been revealed after cleavage in UHV [18], we carefully examined the positions where additional spots should be expected for a (2 x 2) reconstruction in case of the Ag‐terminated side and a (4 x 5√3)_rect._ for the I‐terminated side. However, no additional spots were found within the reciprocal bulk unit cell upon a more detailed examination of the FFTs of the AFM images. Calculations for UHV conditions also suggest vertical relaxation of the surface atoms [18]. However, such a vertical relaxation cannot unambiguously be identified with the AFM due to the lack of direct comparison with the bulk‐terminated surface. Height profiles also do not give access to this information, as the same vertical relaxation is expected to take place on all terraces. Thus, our AFM images do not provide any evidence of surface reconstruction at the atomic scale in any of the investigated systems. This finding is consistent with our previous AFM measurements at the AgI–water and the AgI–ethanol interface [25], in which both surfaces have also been found to exhibit a stable bulk‐truncated structure at the atomic scale throughout long‐term measurements.

Interestingly, it is evident from Figure 2 that the corrugation seen in the images of the I‐terminated planes is more pronounced than that in the images of the Ag‐terminated planes. This trend was observed for all eleven high‐resolution measurements, in which both planes of the same sample were investigated. At this point, we do not yet have a conclusive explanation for this observation, but it could be an indication of a more stable nature of the I‐terminated as compared to the Ag‐terminated plane. While conducting experiments at the AgI‐liquid interfaces, we found that resolving a periodic structure on the atomic scale proved to be more challenging compared to other extensively studied substrates, such as calcite, where high‐resolution images can typically be captured reliably even during long‐term measurements. This difficulty may be attributed to the intrinsic properties and instability of the polar AgI surfaces; however, further investigation is needed to confirm this.

### 3.2 Large‐Scale Images in Pure Water

**FIGURE 3:**
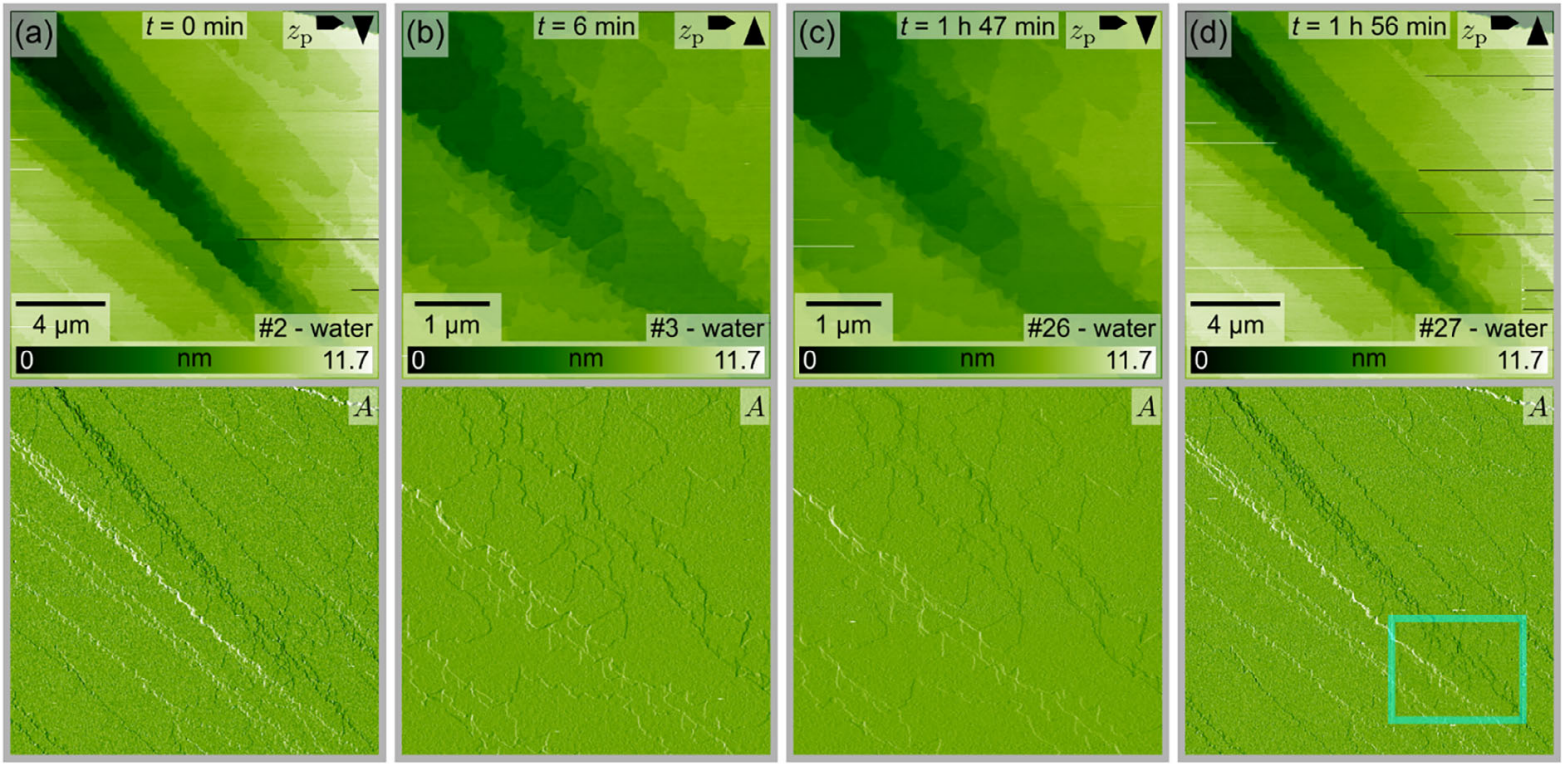
Selected AFM images (height channel *z*_*p*_ and corresponding amplitude *A* images) from a series taken at the Ag‐terminated AgI(0001)‐water interface. The time *t* indicates the elapsed time between the images shown in (b–d) relative to the image in (a). The cyan frame in (d) marks approximately the previous scanning area. Due to the instrument's overscanning, the scanning area is wider than the images in (b,c). The image number in the series is shown in the lower right corner, and the arrows in the upper right corner indicate the fast and slow scan directions. A video file “F3_water(0001)Ag.mp4” corresponding to this image series is provided in the Supporting Information.

**FIGURE 4:**
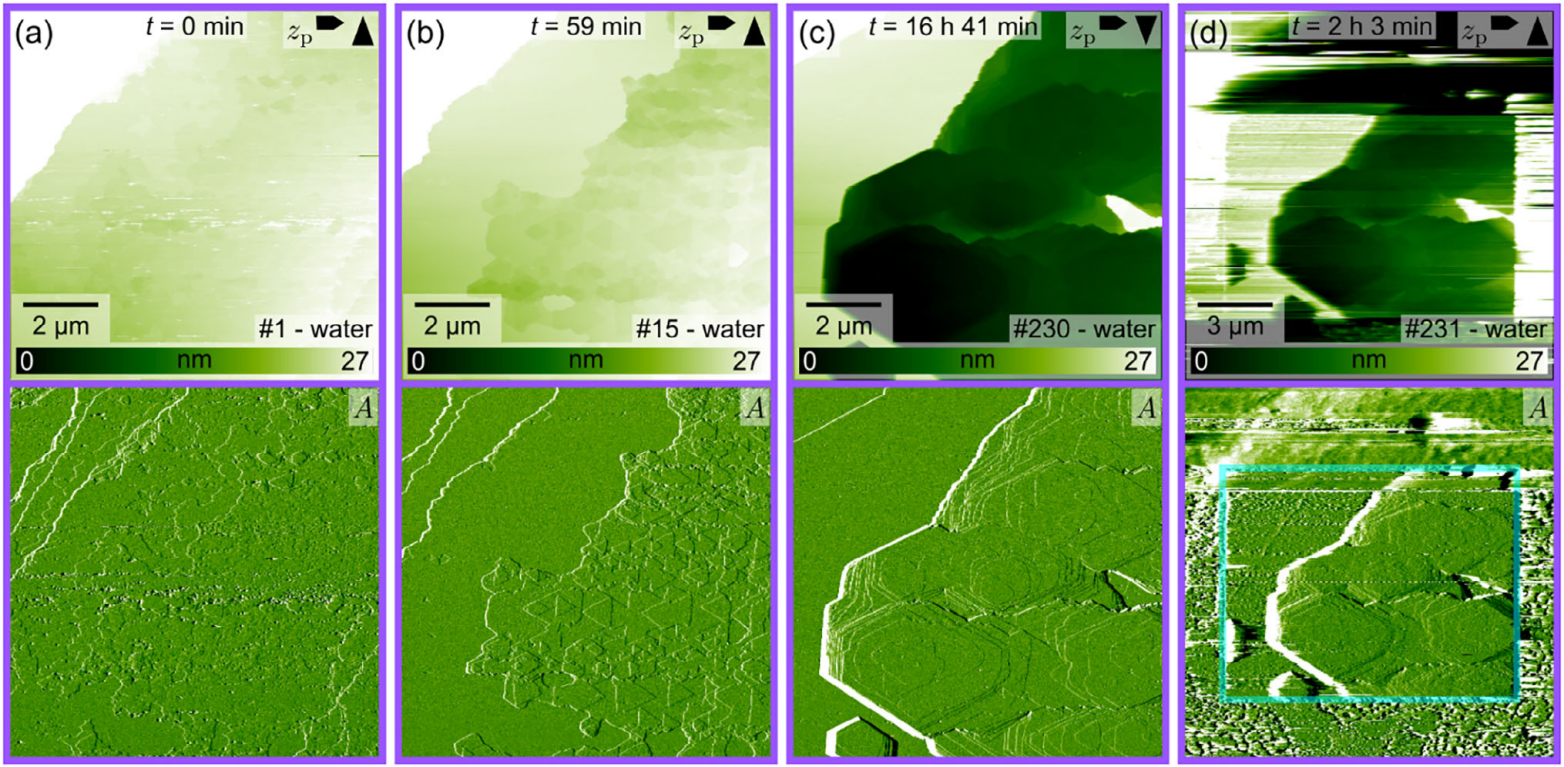
Selected AFM images (height channel *z*_p_ and corresponding amplitude *A* images) from a series taken at the I‐terminated AgI(000–1)‐water interface. The time *t* indicates the elapsed time between the images shown in (b)–(d) relative to the image in (a). The cyan frame in (d) marks approximately the previous scanning area. Due to the instrument's overscanning, the scanning area is wider than the images in (a)–(c). The image number in the series is shown in the lower right corner, and the arrows in the upper right corner indicate the fast and slow scan directions. A video file “F4_water(000–1)I.mp4” corresponding to this image series is provided in the Supporting Information.

With a solubility product constant of 8.52·10^−17^ at 25°C [33], AgI is practically insoluble in water. In the following, we show selected micrometer‐scale AFM images recorded at the interface of the Ag‐terminated (Figure 3) and the I‐terminated plane (Figure 4) in pure water. For both surface terminations, only minor changes in the surface structure can be seen. The most prominent changes seem to be induced by the scanning process. This is deduced from the zoom‐out images at the end of each series, which typically display a rectangular area of more pronounced changes at the position of the previous scans. In the images of the Ag‐terminated plane in Figure 3, edges and tapered terraces can be seen that are characteristic for an as‐cleaved surface. These surface features are consistently observed throughout the entire imaging series, which lasted approximately 2 h. Figure 3b,c show terrace edges with a saw‐toothed shape, which are characteristic of the hexagonal wurtzite structure [34]. Notably, the density of these edges does not increase, contrary to what one might expect if the edge terminations were involved in a surface stabilization [14]. Comparing Figure 3a,d, the scanning area of the previously taken images can be recognized in the lower right part of the image marked by a cyan rectangle. This image series demonstrates that the Ag‐terminated surface does not change significantly when kept in pure water. In other image series, more pronounced dissolution was sometimes observed in the scanned area. However, also in these instances, no significant changes were detected in regions outside the scanning area. For the (0001)‐Ag plane, the scanning‐induced dissolution typically proceeded along the edges of triangular etch pits, resulting in a reduction rather than an increase of the step edge density in the scanning area compared to the regions outside. This is an unexpected finding, considering that the formation of I‐terminated step edges could be a reasonable stabilization mechanism by reducing the surface charge of the polar plane [14]. More image series illustrating the dissolution process induced by the scanning process are shown in the Supporting Information (Section I).

Figure 4 shows selected images from a series taken at the interface of the (000–1)‐I plane in water. The surface exhibits terraces with triangular etch pits. The triangles in successive layers exhibit an alternating orientation, which is characteristic of the wurtzite crystal structure, in which successive layers are rotated by 60° [34]. For the I‐terminated planes, the formation of pits inside already existing etch pits was observed more frequently as compared to the Ag‐terminated plane. As shown in Figure 4a–c, the initial, comparably small triangular pits grew deep and eventually resulted in a single hexagonal pit (Figure 4d). As mentioned above, major changes in the structure only took place in the scanning area, as evident from Figure 4d, where an etch pit is seen in the central rectangular area marked in cyan. Apart from the formation of these pits, stable and very flat areas, as those visible in Figure 4b,c, were formed. In line with our interpretation of the more pronounced corrugation obtained in the atomic‐resolution images of the (000–1)‐I planes in Figure 2, this could be an indication that the I‐terminated surface is more stable than the (0001)‐Ag plane. Additional image series illustrating both stable regions and surface changes within the scanning area on the (000–1)‐I plane are available in the Supporting Information (Section II).

As previous MD simulations have addressed the effect of adding ions [10], we investigated the influence of adding sodium and chloride ions that do not alter the AgI solubility. The trends reported here for both cleavage planes remain unchanged when adding NaCl to the solution. Respective image series in the presence of 0.1 and 1 M NaCl aqueous solutions are presented in the Supporting Information (Section III).

### 3.3 Large‐Scale Images in n‐Dodecane

**FIGURE 5:**
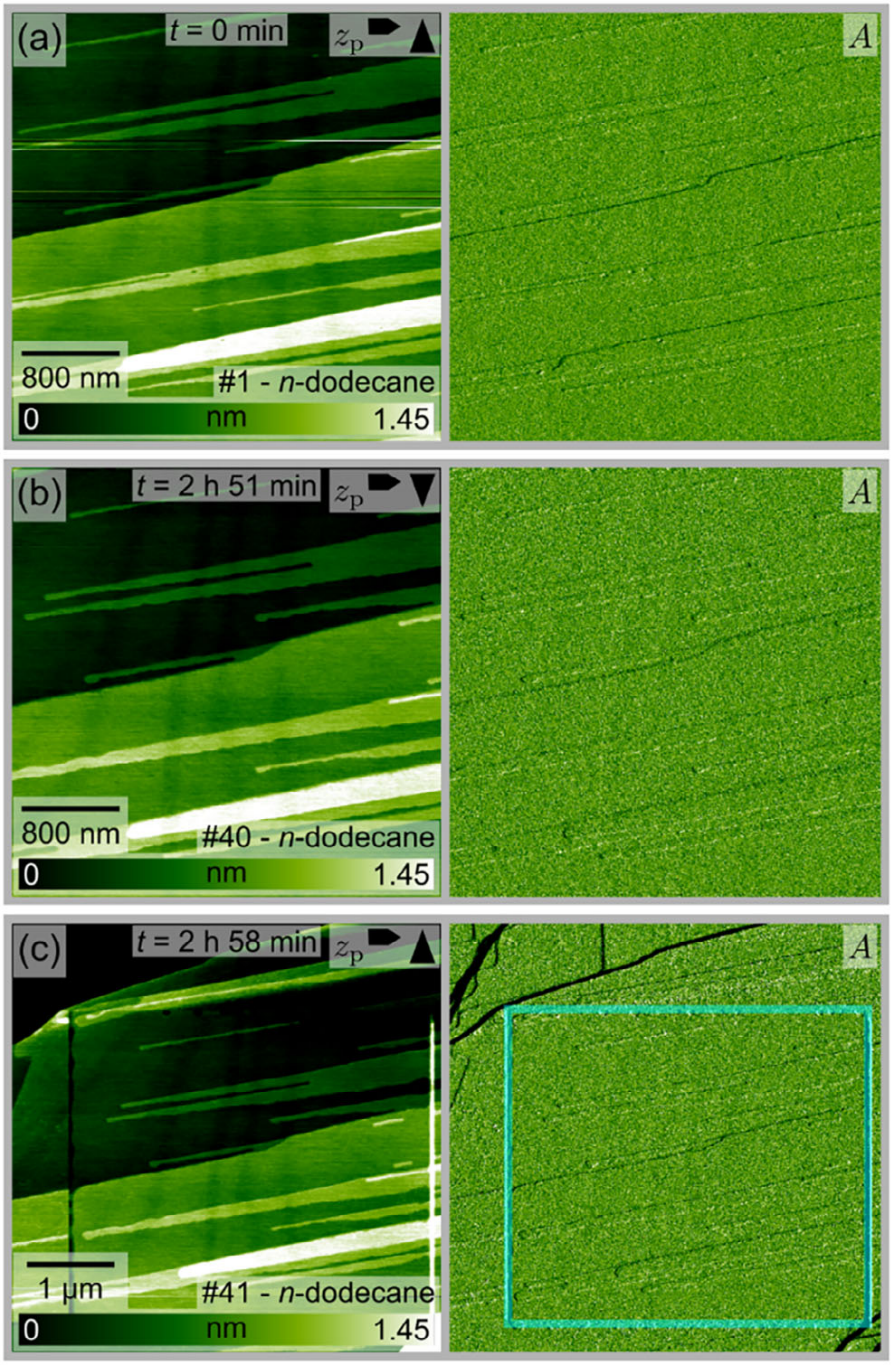
Selected AFM images (height channel *z*_p_ and corresponding amplitude *A* images) from a series taken at the Ag‐terminated AgI(0001)‐*n*‐dodecane interface. The time *t* indicates the elapsed time between the images shown in (b,c) relative to the image in (a). The cyan frame in (c) marks approximately the previous scanning area. Due to the instrument's overscanning, the scanning area is wider than the images in (a,b). The image number in the series is shown in the lower right corner, and the arrows in the upper right corner indicate the fast and slow scan directions. A video file “F5_dodecane(0001)Ag.mp4” corresponding to this image series is provided in the Supporting Information.

As *n*‐dodecane is a non‐polar solvent, AgI is expected to be even less soluble in this solvent than in pure water. Indeed, we observe even less changes in *n*‐dodecane as compared to water. Selected AFM images from a series recorded at the interface of the Ag‐terminated (0001) plane in *n*‐dodecane are shown in Figure 5. In these images, step edges characteristic of a cleaved mineral are seen. Essentially, no changes can be observed during the 3‐h measurement, except for a straight trough that has formed at the turning point of the tip during scanning (Figure 5c).

**FIGURE 6:**
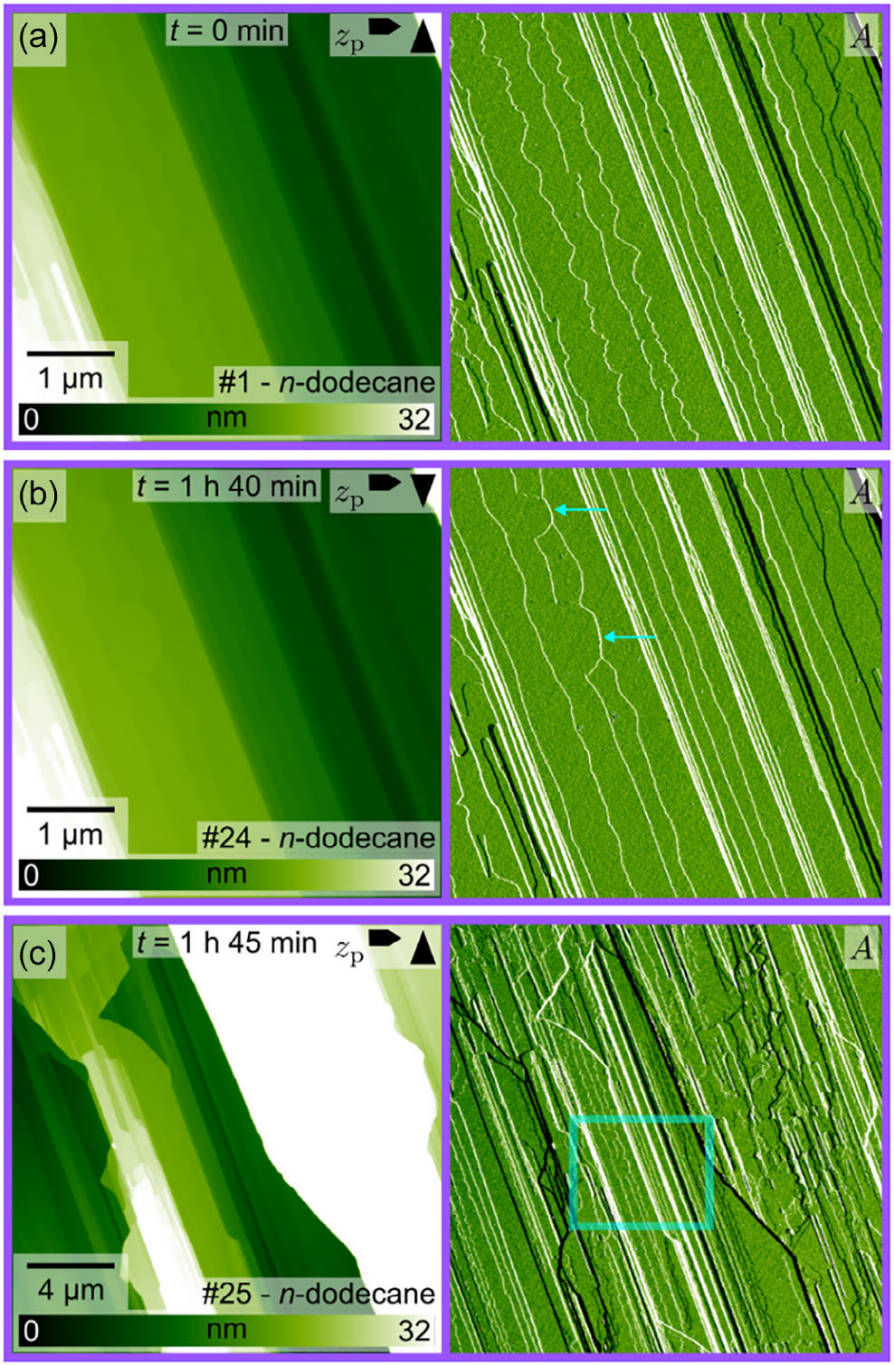
Selected AFM images (height channel *z*_p_ and corresponding amplitude *A* images) from a series taken at the I‐terminated AgI(000–1)‐*n*‐dodecane interface. The cyan arrows in (b) highlight the positions at which two terraces combine. The cyan frame in (c) marks approximately the previous scanning area. Due to the instrument's overscanning, the scanning area is wider than the images in (a,b). The time *t* indicates the elapsed time between the images shown in (b,c) relative to the image in (a). The image number in the series is shown in the lower right corner, and the arrows in the upper right corner indicate the fast and slow scan directions. A video file “F6_dodecane(000–1)I.mp4” corresponding to this image series is provided in the Supporting Information.

A similar picture is obtained for the I‐terminated (000–1) plane in *n*‐dodecane as shown in Figure 6. Here, the as‐cleaved surface reveals a larger number of step edges, which become somewhat straighter during scanning. In one instance, marked by cyan arrows in Figure 6b, two terraces combine, resulting in the formation of less but higher steps. This kind of step ripening was observed for several samples of the (000–1)‐I plane in *n*‐dodecane. In the zoom‐out image in Figure 6c, the initial scan area cannot be recognized, indicating that the I‐terminated surface plane in *n*‐dodecane is not significantly affected by the scanning process.

During AFM experiments conducted in *n*‐dodecane, we occasionally encounter roundish features that cover the surface. As these features are observed to move and coalesce on the surface, we attribute these features to water droplets. These droplets are likely to originate from aqueous inclusions within the AgI crystal, which are released during the cleavage process. It appears plausible that during the growth of the crystal in an aqueous KI solution, a small amount of the solution becomes incorporated within the crystal. Subsequently, upon cleavage in a non‐polar solvent, these inclusions adhere to the polar surface of the crystal and become visible during measurements. To support this interpretation, we present images of the (0001)‐Ag surface taken with a confocal microscope in the Supporting Information (Section IV). These images show macroscopic features on the cleavage plane that we ascribe to an aqueous liquid. The occurrence of an aqueous phase on the Ag‐terminated surface causes the initially stable edges to change until all distinct surface features have vanished. In the Supporting Information (Section V), we present additional image series showcasing the variety of surface features observed at the AgI‐*n*‐dodecane interface.

### 3.4 Large‐Scale Images in 0.1 M KI Aqueous Solution

**FIGURE 7:**
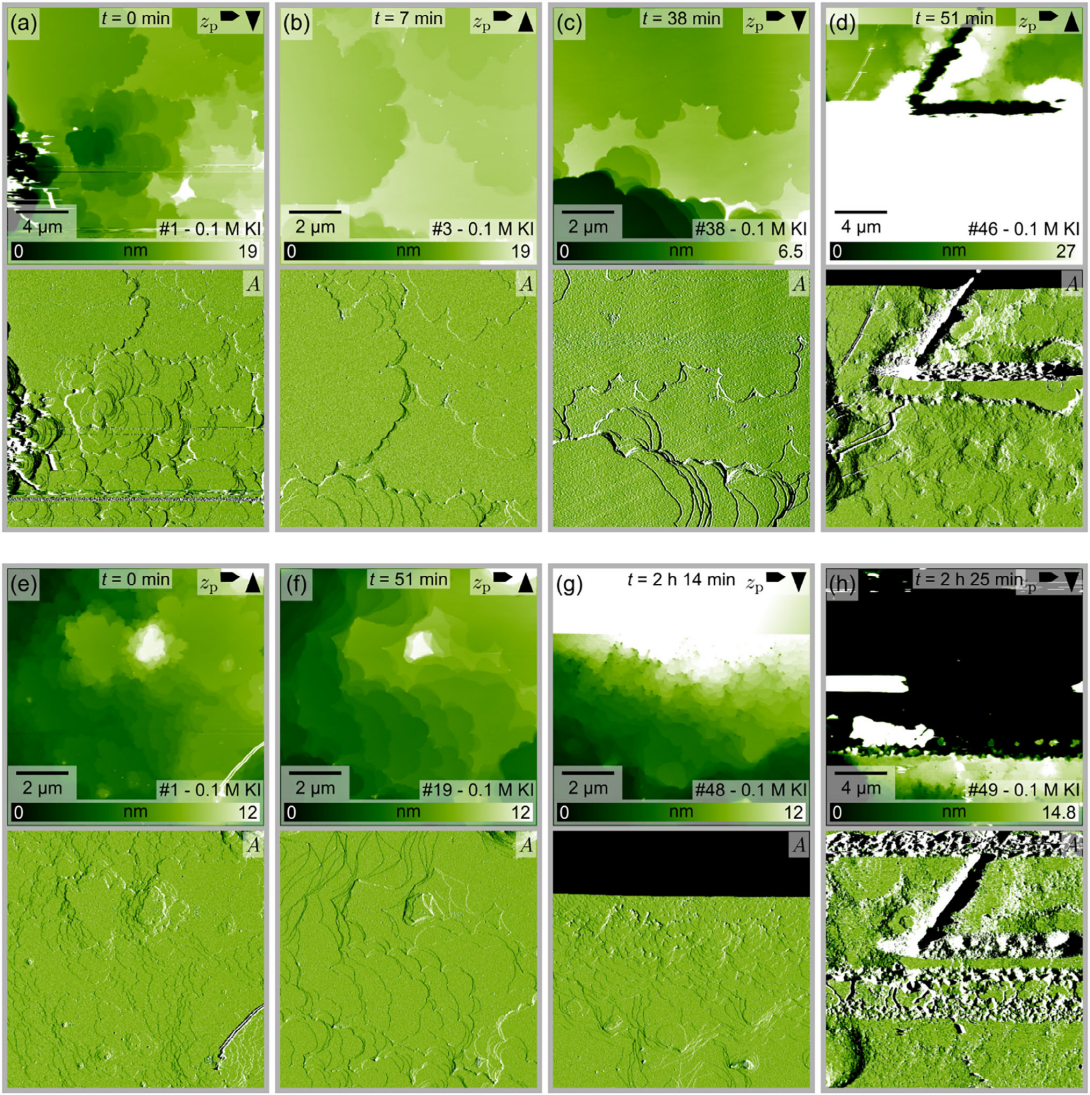
Selected AFM images (height channel *z*_p_ and corresponding amplitude *A* images) from two series ((a–d) and (e–h)) taken at the interface between the Ag‐terminated AgI(0001) plane and an 0.1 M KI aqueous solution. The time *t* indicates the elapsed time between the images shown in (b–d) relative to the image in (a) and in (f–h) relative to the image in (e), respectively. The image number in the series is shown in the lower right corner, and the arrows in the upper right corner indicate the fast and slow scan directions. The video files “F7_ad_01M_KI(0001)Ag.mp4” and “F7_e‐h_01M_KI(0001)Ag.mp4” corresponding to these image series are provided in the Supporting Information.

As silver iodide is practically insoluble in water and *n*‐dodecane, it can be speculated that the surface remains in the thermodynamically unstable, as‐cleaved state only because any rearrangement is prohibited by the low AgI solubility in these solvents. Thus, it is worth considering whether a large‐scale reconstruction might occur in a solvent in which AgI is highly soluble. To this end, we investigated both cleavage planes in a 0.1 M potassium iodide solution. AgI is soluble in iodide‐containing solutions due to the complexation of Ag^+^ cations in [AgI_2_]^−^ [26, 35]. Indeed, image series of the Ag‐terminated plane in Figure 7 show a rapid dissolution of the surface. The pits that grew during this process exhibit a less pronounced triangular shape as compared to measurements in water, but rather rounded and convex edges. However, an alternating orientation of the triangles in successive layers can still be recognized in some areas. A video provided in the Supporting Information (video file “F7_eh_01M_KI(0001)Ag.mp4”) of the image series corresponding to Figure 7e,h further illustrates that the terraces not only dissolve (images with index 62–87 in video file), but also grow (images with index 55–61 in video file).

**FIGURE 8:**
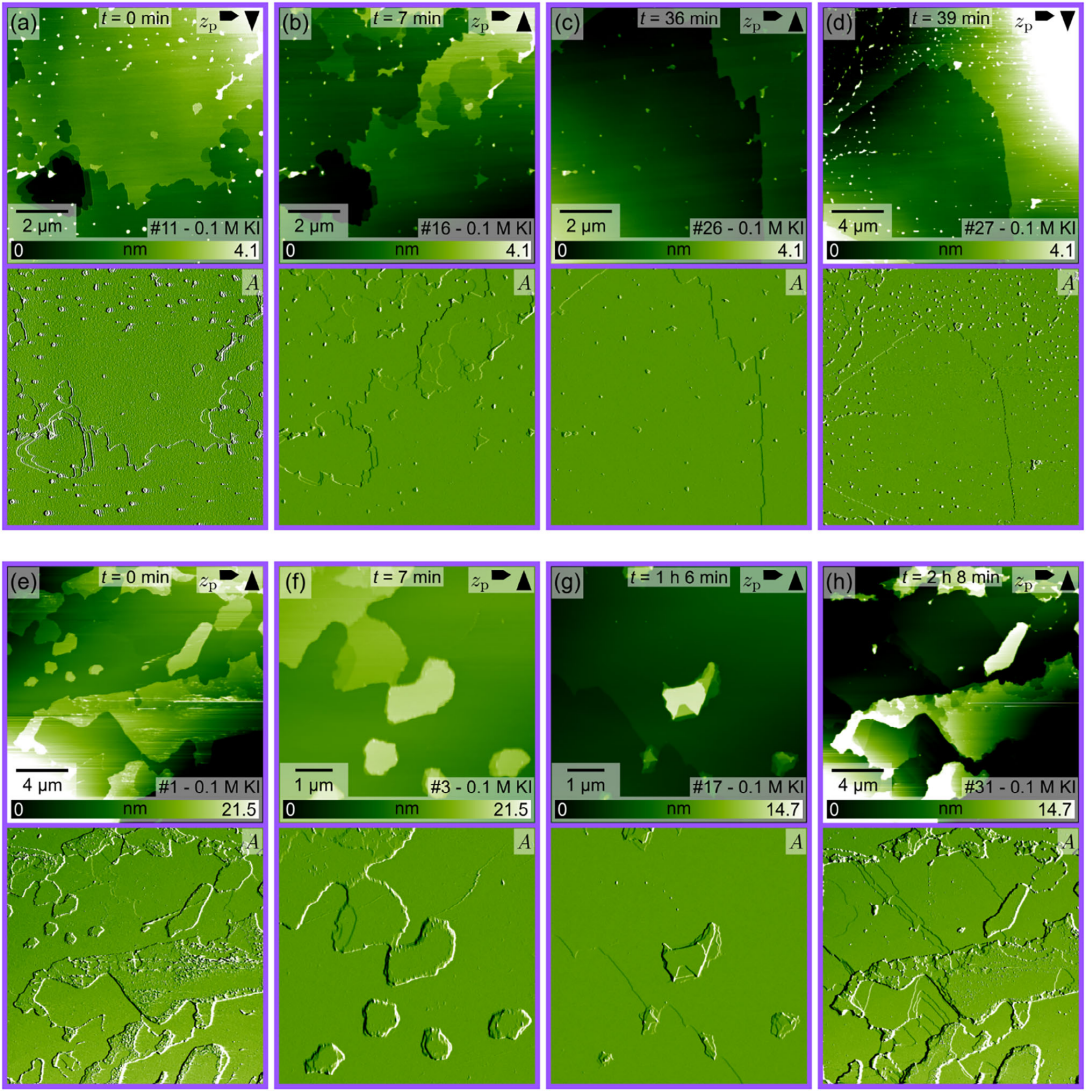
Selected AFM images (height channel *z*_p_ and corresponding amplitude *A* images) from two series ((a–d) and (e–h)) taken at the interface between the I‐terminated AgI(000–1) plane and an 0.1 M KI aqueous solution. The time *t* indicates the elapsed time between the images shown in (b–d) relative to the image in (a) and in (f–h) relative to the image in (e), respectively. The image number in the series is shown in the lower right corner, and the arrows in the upper right corner indicate the fast and slow scan directions. The video files “F8_ad_01M_KI(000–1)I.mp4” and “F8_e‐h_01M_KI(000–1)I.mp4” corresponding to these image series are provided in the Supporting Information.

Dissolution is also observed for the I‐terminated plane in 0.1 M KI aqueous solution (Figure 8). In this case, the triangular shape of growing pits is more pronounced and the reduction of step edges inside the scanning area seems to be more favorable as compared to the Ag‐terminated side. No growth of terraces was observed in these measurements. The series shown in Figure 8a–d clearly illustrates the formation of one large triangular pit. In Figure 8d, a zoom‐out image is given. The previous scanning area in the center of this image differs only marginally from the area outside. The most prominent change in the scanning area is the absence of the small islands that are scattered over the terraces outside the scanning area. Minor changes are also evident in Figure 8e–h, specifically in the upper left corner, where smaller images were captured. In this region, large flat terraces with long and relatively straight edges have formed.

Additional AFM and light microscope images collected for experiments in a 1 M KI (Supporting Information, Section VI) and in a 1 M sodium iodide aqueous solution (NaI) (Supporting Information, Section VII) confirmed the dissolution of AgI in iodide‐containing solutions and provided further indications for the different nature of the two cleavage planes. The optical microscope images of the polar, Ag‐terminated (0001) plane in the Supporting Information show an overgrowth with numerous pyramidal‐shaped structures, causing the exposed (0001) surface area to vanish.

The important finding of the measurements in iodide‐containing solutions is that, even though silver iodide can and does dissolve, no evolution of a triangular reconstruction or any other significant change in the surface morphology toward a large‐scale surface reconstruction seems to take place. All changes observed are characteristic of mineral dissolution and growth as usually seen for minerals in a good solvent. This finding supplements the aforementioned bulk‐truncated structure found on the atomic scale. Interestingly, since no indications of surface reconstruction can be found, the question of how the polar surfaces are stabilized remains open.

## 4 Conclusion

We have investigated the Ag‐terminated (0001) and the I‐terminated (000–1) surfaces of β‐AgI after cleavage in various solvents with different AgI solubility. We cleaved the crystal in the respective solvent and imaged both cleavage planes while keeping the sample immersed in the solvent. We compare the results obtained in pure water, having a poor AgI solubility, with those obtained after cleavage in *n*‐dodecane, featuring an even lower solubility for AgI. To address the influence of added ions, we also investigate samples after cleavage in 0.1 M NaCl aqueous solution. The results obtained with these three solvents are contrasted with experiments carried out in 0.1 KI aqueous solution, having a good solubility for AgI. Regardless of the solvent used, our high‐resolution images consistently reveal atomic resolution that is compatible with the bulk‐truncated structure of the crystal planes. Interestingly, the I‐terminated (000–1) surface is found to be more easily imaged than the Ag‐terminated (0001), indicating a larger corrugation and/or a more stable structure at the (000–1) surface.

Besides the atomic‐scale images, we also collected images at a micrometer scale. At this larger scale, the cleavage planes exhibit a rich variety of different features that appear to depend on the quality of the cleavage, the cleanliness of the given crystal and the surface termination. Besides straight step edges characteristic of cleavage, we observed triangular pits and occasionally other surface features. Despite this variety, several general statements can be made. When cleaved in water, only minor changes can be observed, which aligns with the fact that AgI is poorly soluble in pure water. We observe that surface changes are assisted by the scanning process. However, in areas where changes are observed, the surface appears to become flatter rather than reconstructed. In particular, we do not find an indication for a large‐scale triangular reconstruction, neither on the Ag‐terminated, nor on the I‐terminated side. The situation remains the same when increasing the ion concentration by adding NaCl, which does not alter the solubility of AgI. The situation is similar to what is observed in *n*‐dodecane, confirming the picture that the cleavage planes in water are basically hindered from rearranging due to the poor AgI solubility. However, the addition of iodide ions does change the solubility, as [AgI_2_]^−^ complexes can form in excess of iodide ions. In a 0.1 M KI aqueous solution, we do observe the step edges to retreat and advance in a manner that is characteristic for mineral dissolution and growth. Interestingly, even in this case, in which AgI can and does dissolve, we cannot find an indication for a stabilizing surface reconstruction to evolve. Of course, a reconstruction on the atomic scale could be disordered and, hence, may be so subtle that we are unable to detect it with AFM. Nonetheless, the consistent results observed on both surface terminations and across all solvents do not offer compelling evidence for a periodic or a triangular reconstruction occurring.

## Supporting Information

Additional supporting information can be found online in the Supporting Information section. **Supporting Fig. S1**: AFM images (height channel *z*_p_) as shown in Figure 3a and 3b in the main text, with height profiles extracted along the orange lines. **Supporting Fig. S2**: Selected AFM images (height channel *z*_p_ and corresponding amplitude *A* images) from a series taken at the Ag‐terminated AgI(0001)‐water interface. The height profile shown in (d) was extracted along the orange line. The time *t* indicates the elapsed time between the images shown in (b)–(d) relative to the image in (a). The image number in the series is shown in the lower right corner, and the arrows in the upper right corner indicate the fast and slow scan directions. A video file ‘S2_water(0001)Ag.mp4’ corresponding to this image series is provided in the Supporting Information. **Supporting Fig. S3**: Selected AFM images (height channel *z*_p_ and corresponding amplitude *A* images) from a series taken at the Ag‐terminated AgI(0001)‐water interface. The time *t* indicates the elapsed time between the images shown in (b)–(d) relative to the image in (a). The image number in the series is shown in the lower right corner, and the arrows in the upper right corner indicate the fast and slow scan directions. A video file ‘S3_water(0001)Ag.mp4’ corresponding to this image series is provided in the Supporting Information. **Supporting Fig. S4**: Selected AFM images (height channel *z*_p_ and corresponding amplitude *A* images) from a series taken at the Ag‐terminated AgI(0001)‐water interface. The height profiles shown in (c) and (d) were extracted along the orange lines. The time *t* indicates the elapsed time between the images shown in (b)–(d) relative to the image in (a). The image number in the series is shown in the lower right corner, and the arrows in the upper right corner indicate the fast and slow scan directions. A video file ‘S4_water(0001)Ag.mp4’ corresponding to this image series is provided in the Supporting Information. **Supporting Fig. S5**: Selected AFM images (height channel *z*_p_ and corresponding amplitude *A* images) from a series taken at the Ag‐terminated AgI(0001)‐water interface. The height profiles shown in (c) and (d) were extracted along the orange lines. The time *t* indicates the elapsed time between the images shown in (b)–(d) relative to the image in (a). The image number in the series is shown in the lower right corner, and the arrows in the upper right corner indicate the fast and slow scan directions. A video file ‘S5_water(0001)Ag.mp4’ corresponding to this image series is provided in the Supporting Information. **Supporting Fig. S6**: AFM images (height channel *z*_p_) as shown in Figure 4b and 4d in the main text, with height profiles extracted along the orange lines. **Supporting Fig. S7**: Selected AFM images (height channel *z*_p_ and corresponding amplitude *A* images) from a series taken at the I‐terminated AgI(000−1)‐water interface. The time *t* indicates the elapsed time between the images shown in (b)–(d) relative to the image in (a). The image number in the series is shown in the lower right corner, and the arrows in the upper right corner indicate the fast and slow scan directions. A video file ‘S7_water(000−1)I.mp4’ corresponding to this image series is provided in the Supporting Information. **Supporting Fig. S8**: Selected AFM images (height channel *z*_p_ and corresponding amplitude *A* images) from a series taken at the I‐terminated AgI(000−1)‐water interface. The time *t* indicates the elapsed time between the images shown in (b)–(d) relative to the image in (a). The image number in the series is shown in the lower right corner, and the arrows in the upper right corner indicate the fast and slow scan directions. A video file ‘S8_water(000−1)I.mp4’ corresponding to this image series is provided in the Supporting Information. **Supporting Fig. S9**: Selected AFM images (height channel *z*_p_ and corresponding amplitude *A* images) from a series taken at the interface between the Ag‐terminated AgI(0001) plane and a 0.1 M NaCl aqueous solution. The height profiles shown in (c) and (d) were extracted along the orange lines. The time *t* indicates the elapsed time between the images shown in (b)–(d) relative to the image in (a). The image number in the series is shown in the lower right corner, and the arrows in the upper right corner indicate the fast and slow scan directions. A video file ‘S9_01M_NaCl(0001)Ag.mp4’ corresponding to this image series is provided in the Supporting Information. **Supporting Fig. S10**: Selected AFM images (height channel *z*_p_ and corresponding amplitude *A* images) from a series taken at the interface between the Ag‐terminated AgI(0001) plane and a 0.1 M NaCl aqueous solution. The height profiles shown in (c) and (d) were extracted along the orange lines. As shown in (d), a deep hole is formed during scanning. The pronounced hole formation is tentatively ascribed to the long scanning time of this series rather than the presence of NaCl. The time *t* indicates the elapsed time between the images shown in (b)–(d) relative to the image in (a). The image number in the series is shown in the lower right corner, and the arrows in the upper right corner indicate the fast and slow scan directions. A video file ‘S10_01M_NaCl(0001)Ag.mp4’ corresponding to this image series is provided in the Supporting Information. **Supporting Fig. S11**: Selected AFM images (height channel *z*_p_ and corresponding amplitude *A* images) from a series taken at the interface between the Ag‐terminated AgI(0001) plane and a 1 M NaCl aqueous solution. The height profiles shown in (c) and (d) were extracted along the orange lines. The time *t* indicates the elapsed time between the images shown in (b)–(d) relative to the image in (a). The image number in the series is shown in the lower right corner, and the arrows in the upper right corner indicate the fast and slow scan directions. A video file ‘S11_1M_NaCl(0001)Ag.mp4’ corresponding to this image series is provided in the Supporting Information. **Supporting Fig. S12**: Selected AFM images (height channel *z*_p_ and corresponding amplitude *A* images) from a series taken at the interface between the I‐terminated AgI(000−1) plane and a 0.1 M NaCl aqueous solution. The height profile shown in (d) was extracted along the orange line. The time *t* indicates the elapsed time between the images shown in (b)–(d) relative to the image in (a). The image number in the series is shown in the lower right corner, and the arrows in the upper right corner indicate the fast and slow scan directions. A video file ‘S12_0.1M_NaCl(000−1)I.mp4’ corresponding to this image series is provided in the Supporting Information. **Supporting Fig. S13**: Selected AFM images (height channel *z*_p_ and corresponding amplitude *A* images) from a series taken at the interface between the I‐terminated AgI(000−1) plane and a 1 M NaCl aqueous solution. The height profiles shown in (c) and (d) were extracted along the orange lines. The time *t* indicates the elapsed time between the images shown in (b)–(d) relative to the image in (a). The image number in the series is shown in the lower right corner, and the arrows in the upper right corner indicate the fast and slow scan directions. A video file ‘S13_1M_NaCl(000−1)I.mp4’ corresponding to this image series is provided in the Supporting Information. **Supporting Fig. S14**: Images of two cleaved AgI samples taken with a DCM8 optical microscope (Leica Microsystems GmbH, Germany) in a confocal mode. a) Favorable AgI(0001)‐Ag cleavage plane for AFM experiments, without macroscopically visible impurities. 50‐fold magnification. Brightness and contrast were increased by 30% and 10%, respectively. b) AgI(0001)‐Ag cleavage plane with macroscopically visible droplets and impurities. Ten‐fold magnification. Brightness was increased by 10%. **Supporting Fig. S15**: AFM image (height channel *z*_p_) as shown in Figure 5c in the main text, with a height profile extracted along the orange line. **Supporting Fig. S16**: Selected AFM images (height channel *z*_p_ and corresponding amplitude *A* images) from a series taken at the Ag‐terminated AgI(0001)‐*n*‐dodecane interface. The time *t* indicates the elapsed time between the images shown in (b)–(d) relative to the image in (a). The image number in the series is shown in the lower right corner, and the arrows in the upper right corner indicate the fast and slow scan directions. A video file ‘S16_dodecane(0001)Ag.mp4’ corresponding to this image series is provided in the Supporting Information. **Supporting Fig. S17**: Selected AFM images (height channel *z*_p_ and corresponding amplitude *A* images) from a series taken at the Ag‐terminated AgI(0001)‐*n*‐dodecane interface. The time *t* indicates the elapsed time between the images shown in (b)–(d) relative to the image in (a). The image number in the series is shown in the lower right corner, and the arrows in the upper right corner indicate the fast and slow scan directions. A video file ‘S17_dodecane(0001)Ag.mp4’ corresponding to this image series is provided in the Supporting Information. **Supporting Fig. S18**: Selected AFM images (height channel *z*_p_ and corresponding amplitude *A* images) from a series taken at the Ag‐terminated AgI(0001)‐*n*‐dodecane interface. The height profile shown in (a) was extracted along the orange line. The time *t* indicates the elapsed time between the images shown in (b)–(d) relative to the image in (a). The image number in the series is shown in the lower right corner, and the arrows in the upper right corner indicate the fast and slow scan directions. The *z*_p_‐images in (c) and (d) could not be leveled by fitting a plane through three points. A video file ‘S18_dodecane(0001)Ag.mp4’ corresponding to this image series is provided in the Supporting Information. **Supporting Fig. S19**: Selected AFM images (height channel *z*_p_ and corresponding amplitude *A* images) from a series taken at the Ag‐terminated AgI(0001)‐*n*‐dodecane interface. Between the measurements of the images in (c) and (d), atomic‐scale measurements were performed which led to the distinctive feature visible in (d). The time *t* indicates the elapsed time between the images shown in (b)–(d) relative to the image in (a). The image number in the series is shown in the lower right corner, and the arrows in the upper right corner indicate the fast and slow scan directions. A video file ‘S19_dodecane(0001)Ag.mp4’ corresponding to this image series is provided in the Supporting Information. **Supporting Fig. S20**: Selected AFM images (height channel *z*_p_ and corresponding amplitude *A* images) from a series taken at the Ag‐terminated AgI(0001)‐*n*‐dodecane interface. The time *t* indicates the elapsed time between the images shown in (b)–(d) relative to the image in (a). The image number in the series is shown in the lower right corner, and the arrows in the upper right corner indicate the fast and slow scan directions. Only the *z*_p_‐image in (a) could be leveled by fitting a plane through three points. A video file ‘S20_dodecane(0001)Ag.mp4’ corresponding to this image series is provided in the Supporting Information. **Supporting Fig. S21**: AFM image (height channel *z*_p_) as shown in Figure 6b in the main text, with a height profile extracted along the orange line. **Supporting Fig. S22**: Selected AFM images (height channel *z*_p_ and corresponding amplitude *A* images) from a series taken at the I‐terminated AgI(000−1)‐*n*‐dodecane interface. Compared to the image in (c), the image in (d) has an *xy*‐offset. Between the measurements of those two images, atomic‐scale measurements were performed which led to the distinctive feature visible in the lower right corner in (d). The height profiles shown in (a), (b) and (d) were extracted along the orange lines. The time *t* indicates the elapsed time between the images shown in (b)–(d) relative to the image in (a). The image number in the series is shown in the lower right corner, and the arrows in the upper right corner indicate the fast and slow scan directions. A video file ‘S22_dodecane(000−1)I.mp4’ corresponding to this image series is provided in the Supporting Information. **Supporting Fig. S23**: Selected AFM images (height channel *z*_p_ and corresponding amplitude *A* images) from a series taken at the I‐terminated AgI(000−1)‐*n*‐dodecane interface. The time *t* indicates the elapsed time between the images shown in (b)–(d) relative to the image in (a). The image number in the series is shown in the lower right corner, and the arrows in the upper right corner indicate the fast and slow scan directions. A video file ‘S23_dodecane(000−1)I.mp4’ corresponding to this image series is provided in the Supporting Information. **Supporting Fig. S24**: Selected AFM images (height channel *z*_p_ and corresponding amplitude *A* images) from a series taken at the I‐terminated AgI(000−1)‐*n*‐dodecane interface. The time *t* indicates the elapsed time between the images shown in (b)–(d) relative to the image in (a). The image number in the series is shown in the lower right corner, and the arrows in the upper right corner indicate the fast and slow scan directions. A video file ‘S24_dodecane(000−1)I.mp4’ corresponding to this image series is provided in the Supporting Information. **Supporting Fig. S25**: AFM images (height channel *z*_p_) as shown in Figure 7c and 7f in the main text, with height profiles extracted along the orange lines. **Supporting Fig. S26**: AFM images (height channel *z*_p_) as shown in Figure 7c and 7f in the main text, with height profiles extracted along the orange lines. **Supporting Fig. S27**: Images of the AgI cleavage planes in a 1 M KI aqueous solution. (a) AFM image (height channel *z*_p_ and corresponding amplitude *A* image) of the Ag‐terminated AgI(0001) plane. (b) Images of the Ag‐terminated AgI(0001) surface taken with an optical microscope during the AFM experiment. (c) AFM image (height channel *z*_p_ and corresponding amplitude *A* image) of the I‐terminated AgI(000−1) plane. (d) Images of the I‐terminated AgI(000−1) surface taken with an optical microscope during the AFM experiment. The height profiles shown in (a) and (c) were extracted along the orange lines. The arrows in the upper right corner indicate the fast and slow scan directions and the time *t* indicates the elapsed time between the lower image relative to the upper image shown in (b) and (d), respectively. **Supporting Fig. S28**: Images of the AgI cleavage planes in a 1 M NaI aqueous solution. (a) AFM image (height channel *z*_p_ and corresponding amplitude *A* image) of the Ag‐terminated AgI(0001) plane. (b) Images of the Ag‐terminated AgI(0001) surface taken with an optical microscope during the AFM experiment. (c) AFM image (height channel *z*_p_ and corresponding amplitude *A* image) of the I‐terminated AgI(000−1) plane. (d) Images of the I‐terminated AgI(000−1) surface taken with an optical microscope during the AFM experiment. The height profiles shown in (a) and (c) were extracted along the orange lines. The arrows in the upper right corner indicate the fast and slow scan directions and the time *t* indicates the elapsed time between the lower image relative to the upper image shown in (b) and (d), respectively.

## Funding

This study was supported by Deutsche Forschungsgemeinschaft (KU 1980/18−1, INST 215/635−1 FUGG).

## Conflicts of Interest

The authors declare no conflicts of interest.

## Supporting information

Supplementary Material

## Data Availability

The data that support the findings of this study are available in the supplementary material of this article.

## References

[1] H. Pruppacher and J. Klett , Microphysics of Clouds and Precipitation (Springer Dordrecht, 1997).

[2] T. Koop , “Homogeneous Ice Nucleation in Water and Aqueous Solutions,” Zeitschrift für Physikalische Chemie 218 (2004): 1231–1258.

[3] C. Marcolli , B. Nagare , A. Welti , and U. Lohmann , “Ice Nucleation Efficiency of AgI: Review and New Insights,” Atmospheric Chemistry and Physics 16 (2016): 8915–8937.

[4] B. Vonnegut , “The Nucleation of Ice Formation by Silver Iodide,” Journal of Applied Physics 18 (1947): 593–595.

[5] A. J. Miller , C. Fuchs , F. Ramelli , et al., “Quantified Ice‐Nucleating Ability of AgI‐Containing Seeding Particles in Natural Clouds,” Atmospheric Chemistry and Physics 25 (2025): 5387–5407.

[6] M. Fitzner , P. Pedevilla , and A. Michaelides , “Predicting Heterogeneous Ice Nucleation with a Data‐Driven Approach,” Nature Communications 11 (2020): 4777. 10.1038/s41467-020-18605-3 PMC7509812 32963232

[7] P. W. Tasker , “Stability of Ionic‐Crystal Surfaces,” Journal of Physics C: Solid State Physics 12 (1979): 4977–4984.

[8] C. Noguera , “Polar Oxide Surfaces,” Journal of Physics: Condensed Matter 12 (2000): R367–R410.

[9] G. Fraux and J. P. K. Doye , “Note: Heterogeneous Ice Nucleation on Silver‐Iodide‐Like Surfaces,” The Journal of Chemical Physics 141 (2014): 216101. 25481172 10.1063/1.4902382

[10] T. Sayer and S. J. Cox , “Stabilization of AgI's Polar Surfaces by the Aqueous Environment, and Its Implications for Ice Formation,” Physical Chemistry Chemical Physics 21 (2019): 14546–14555. 31204767 10.1039/c9cp02193k

[11] S. A. Zielke , A. K. Bertram , and G. N. Patey , “A Molecular Mechanism of Ice Nucleation on Model AgI Surfaces,” The Journal of Physical Chemistry B 119 (2015): 9049–9055. 25255062 10.1021/jp508601s

[12] S. A. Zielke , A. K. Bertram , and G. N. Patey , “Simulations of Ice Nucleation by Model AgI Disks and Plates,” The Journal of Physical Chemistry B 120 (2016): 2291–2299. 26878341 10.1021/acs.jpcb.5b06605

[13] J. V. Lauritsen , S. Porsgaard , M. K. Rasmussen , et al., “Stabilization Principles for Polar Surfaces of ZnO,” ACS Nano 5 (2011): 5987–5994. 21671628 10.1021/nn2017606

[14] O. Dulub , U. Diebold , and G. Kresse , “Novel Stabilization Mechanism on Polar Surfaces: ZnO(0001)‐Zn,” Physical Review Letters 90 (2003): 016102. 12570628 10.1103/PhysRevLett.90.016102

[15] S. Torbrügge , F. Ostendorf , and M. Reichling , “Stabilization of Zinc‐Terminated ZnO0001 by a Modified Surface Stoichiometry,” The Journal of Physical Chemistry C 113 (2009): 4909–4914.

[16] A. Wander , F. Schedin , P. Steadman , et al., “Stability of Polar Oxide Surfaces,” Physical Review Letters 86 (2001): 3811–3814. 11329330 10.1103/PhysRevLett.86.3811

[17] D. Mora‐Fonz , T. Lazauskas , M. R. Farrow , C. R. A. Catlow , S. M. Woodley , and A. A. Sokol , “Why Are Polar Surfaces of ZnO Stable?,” Chemistry of Materials : A Publication of the American Chemical Society 29 (2017): 5306–5320.

[18] J. I. Hütner , A. Conti , D. Kugler , et al., “Surface Reconstructions Govern Ice Nucleation on Silver Iodide,” Science Advances 11 (2025): eaea2378. 41171903 10.1126/sciadv.aea2378 PMC12577688

[19] B. Glatz and S. Sarupria , “Heterogeneous Ice Nucleation: Interplay of Surface Properties and Their Impact on Water Orientations,” Langmuir 34 (2018):1190–1198. 29020452 10.1021/acs.langmuir.7b02859

[20] B. Glatz and S. Sarupria , “The Surface Charge Distribution Affects the Ice Nucleating Efficiency of Silver Iodide,” The Journal of Chemical Physics 145 (2016): 211924. 28799343 10.1063/1.4966018

[21] A. Kiselev , F. Bachmann , P. Pedevilla , et al., “Active Sites in Heterogeneous Ice Nucleation—the Example of K‐Rich Feldspars,” Science 355 (2017): 367–371. 27940582 10.1126/science.aai8034

[22] M. A. Holden , T. F. Whale , M. D. Tarn , et al., “High‐Speed Imaging of Ice Nucleation in Water Proves the Existence of Active Sites,” Science Advances 5 (2019): eaav4316. 30746490 10.1126/sciadv.aav4316 PMC6358314

[23] G. Roudsari , B. Reischl , O. H. Pakarinen , and H. Vehkamäki , “Atomistic Simulation of Ice Nucleation on Silver Iodide, 0001 Surfaces with Defects,” The Journal of Physical Chemistry C 124 (2020): 436–445.

[24] Y. Yu , M. Chen , Y. Lei , and H. Niu , “Unconventional Ice Nucleation Pathway Induced by Irregular Silver Iodide Surfaces,” Communications Physics 8 (2025): 7.

[25] F. Sabath , C. Aleff , A. Latus , R. Bechstein , and A. Kühnle , “Atomic‐Resolution Imaging of the Ice Nucleating Silver Iodide Surface: Does This Polar Surface Reconstruct at the Atomic Scale?,” Advanced Materials Interfaces 9 (2022): 2201065.

[26] G. Cochrane , “Preparation of Single Crystals of Hexagonal Silver Iodide,” British Journal of Applied Physics 18 (1967): 687–688.

[27] D. R. Mills , C. M. Perrott , and N. H. Fletcher , “The Production of Single Crystals of AgI,” Journal of Crystal Growth 6 (1970): 266–268.

[28] A. N. Mariano and R. E. Hanneman , “Crystallographic Polarity of ZnO Crystals,” Journal of Applied Physics 34 (1963): 384–388.

[29] E. C. Y. Inn , “Photolytic Inactivation of Ice‐Forming Silver Iodide Nuclei,” Bulletin of the American Meteorological Society 32 (1951): 132.

[30] G. W. Bryant and B. J. Mason , “Photolytic De‐Activation of Silver Iodide as an Ice‐Forming Nucleus,” Quarterly Journal of the Royal Meteorological Society 86 (1960): 354–357.

[31] H. Adam , S. Rode , M. Schreiber , K. Kobayashi , H. Yamada , and A. Kühnle , “Photothermal Excitation Setup for a Modified Commercial Atomic Force Microscope,” The Review of Scientific Instruments 85 (2014): 023703. 24593367 10.1063/1.4864084

[32] T. Dickbreder , F. Sabath , L. Höltkemeier , R. Bechstein , and A. Kühnle , “UnDrift: A Versatile Software for Fast Offline SPM Image Drift Correction,” Beilstein Journal of Nanotechnology 14 (2023): 1225–1237. 38170148 10.3762/bjnano.14.101 PMC10760460

[33] M. Haynes , CRC Handbook of Chemistry and Physics, edited by W. M. Haynes (CRC Press (Taylor & Francis Group), 2016) p.2704.

[34] I. Aroyo , International Tables for Crystallography Volume A: Space‐Group Symmetry, edited by M. I. Aroyo (International Union of Crystallography, 2016).

[35] C. H. Gammons and Y. Yu , “The Stability of Aqueous Silver Bromide and Iodide Complexes at 25°C–300°C: Experiments, Theory and Geologic Applications,” Chemical Geology 137 (1997): 155–173.

