# Supplementary material for: Surface Structure of Polar Silver Iodide (0001) in Various Liquids: No Indication for a Surface Reconstruction
Source: Chemphyschem. 2026 Apr 19;27(7):e202500904. doi: 10.1002/cphc.202500904 (PMC13092326; doi:10.1002/cphc.202500904)
Supplement: Supplementary file 1 — Supplementary Material [file CPHC-27-e202500904-s001.zip › cphc70344-sup-0001-SuppData-S1.pdf]

Supporting Information:

Surface Structure of Polar Silver Iodide (0001) in Various Liquids: No Indication for a Surface Reconstruction

Kim N. Dreier,[a] *Annamaria Latus,*[a] *Ralf Bechstein,*[a] *Angelika Kühnle*[a*]

[a]Physical Chemistry I, Faculty of Chemistry, Bielefeld University, Universitätsstraße 25,

33615 Bielefeld, Germany

* angelika.kuehnle@uni-bielefeld.de

Table of Contents

Section I Further image series and height profiles of Ag-terminated (0001) surfaces cleaved in pure water

Section II Further image series and height profiles of I-terminated (000-1) surfaces cleaved in pure water

Section III Image series and height profiles of Ag-terminated (0001) and I-terminated (000-1) surfaces cleaved in 0.1 M and 1 M NaCl aqueous solutions

Section IV Confocal microscopy images of Ag-terminated (0001) surfaces

Section V Further image series and height profiles of Ag-terminated (0001) and I-terminated (000-1) surfaces cleaved in *n*-dodecane

Section VI Images and height profiles of Ag-terminated (0001) and I-terminated (000-1) surfaces cleaved in 0.1 M and 1 M KI aqueous solution

Section VII Images and height profiles of Ag-terminated (0001) and I-terminated (000-1) surfaces cleaved in 1 M NaI aqueous solution

Section I: Further image series and height profiles of Ag-terminated (0001) surfaces cleaved in pure water

Unless otherwise stated in the figure caption, the AFM data was processed as follows. The large-scale AM topography (z_p channel) images were levelled by a mean plane subtraction and fitting a plane through three points. Rows were aligned by a median of differences.

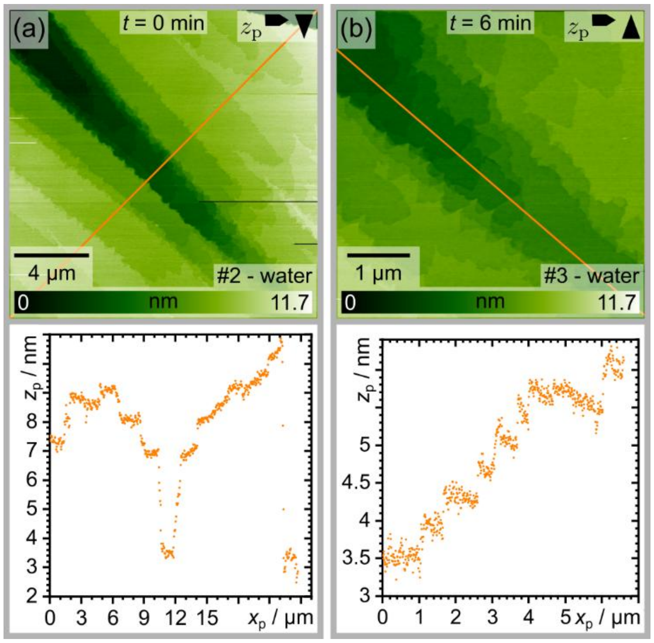

Figure S1. AFM images (height channel z_p) as shown in **Figure 3a** and **3b** in the main text, with height profiles extracted along the orange lines.

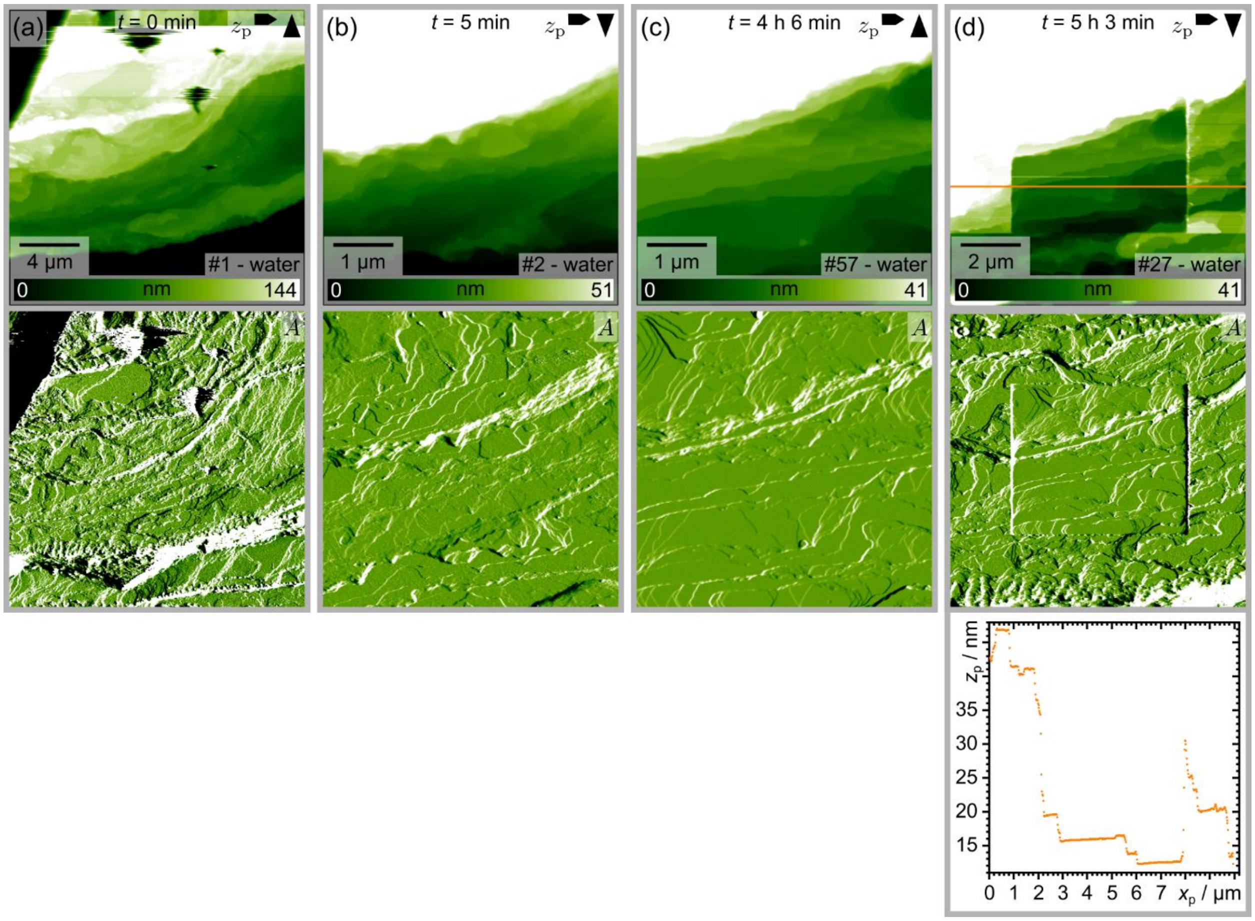

Figure S2. Selected AFM images (height channel z_p and corresponding amplitude A images) from a series taken at the Ag-terminated AgI(0001)-water interface. The height profile shown in (d) was extracted along the orange line. The time t indicates the elapsed time between the images shown in (b) – (d) relative to the image in (a). The image number in the series is shown in the lower right corner, and the arrows in the upper right corner indicate the fast and slow scan directions. A video file "S2_water(0001)Ag.mp4" corresponding to this image series is provided in the Supporting Information.

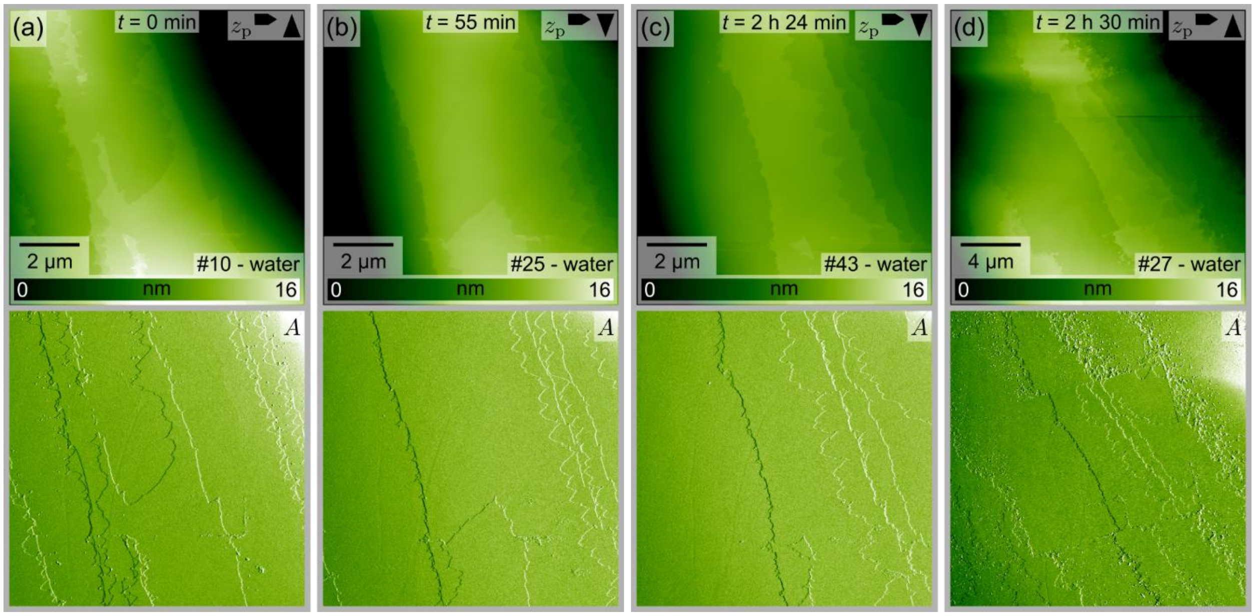

Figure S3. Selected AFM images (height channel z_p and corresponding amplitude A images) from a series taken at the Ag-terminated AgI(0001)-water interface. The time t indicates the elapsed time between the images shown in (b) – (d) relative to the image in (a). The image number in the series is shown in the lower right corner, and the arrows in the upper right corner indicate the fast and slow scan directions. A video file “S3_water(0001)Ag.mp4” corresponding to this image series is provided in the Supporting Information.

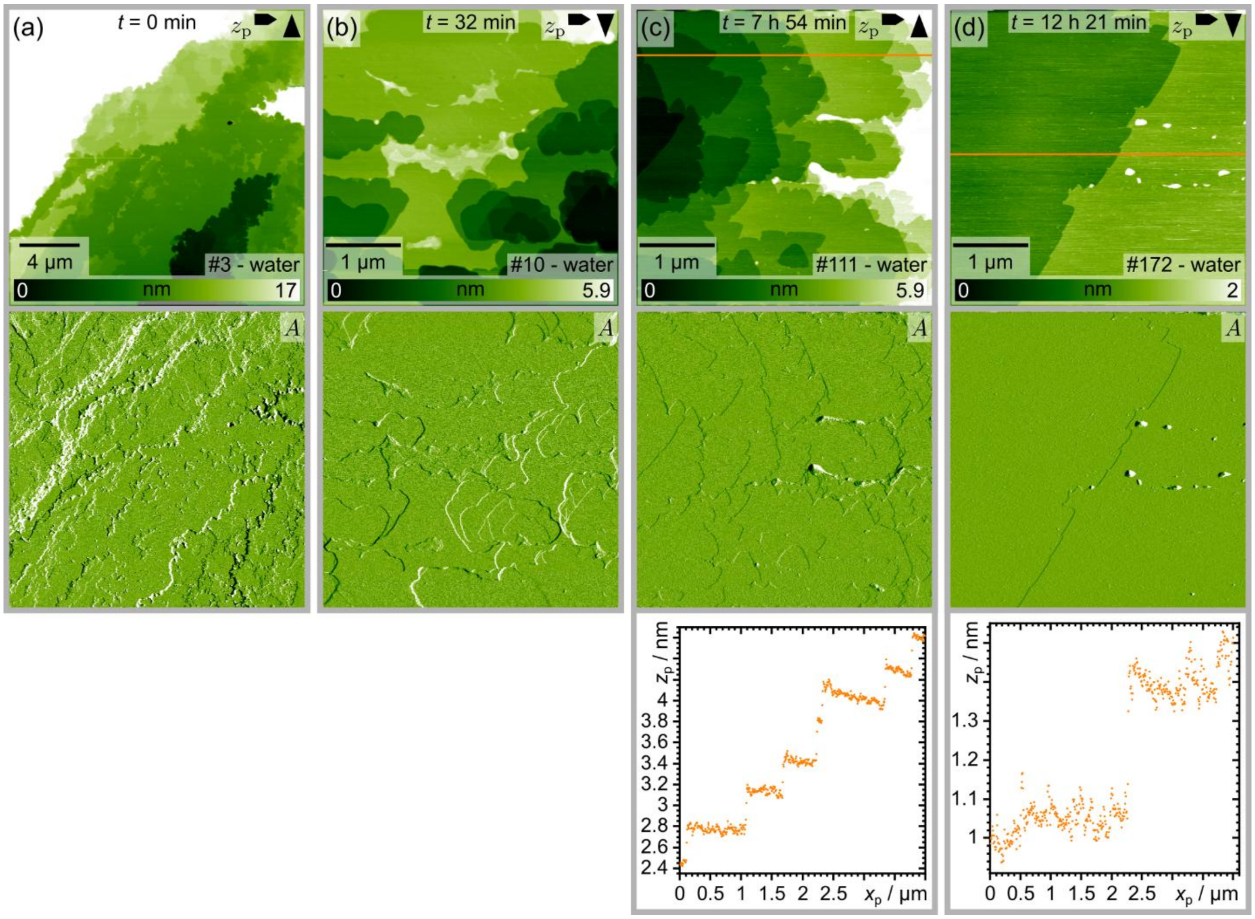

Figure S4. Selected AFM images (height channel z_p and corresponding amplitude A images) from a series taken at the Ag-terminated AgI(0001)-water interface. The height profiles shown in (c) and (d) were extracted along the orange lines. The time t indicates the elapsed time between the images shown in (b) – (d) relative to the image in (a). The image number in the series is shown in the lower right corner, and the arrows in the upper right corner indicate the fast and slow scan directions. A video file "S4_water(0001)Ag.mp4" corresponding to this image series is provided in the Supporting Information.

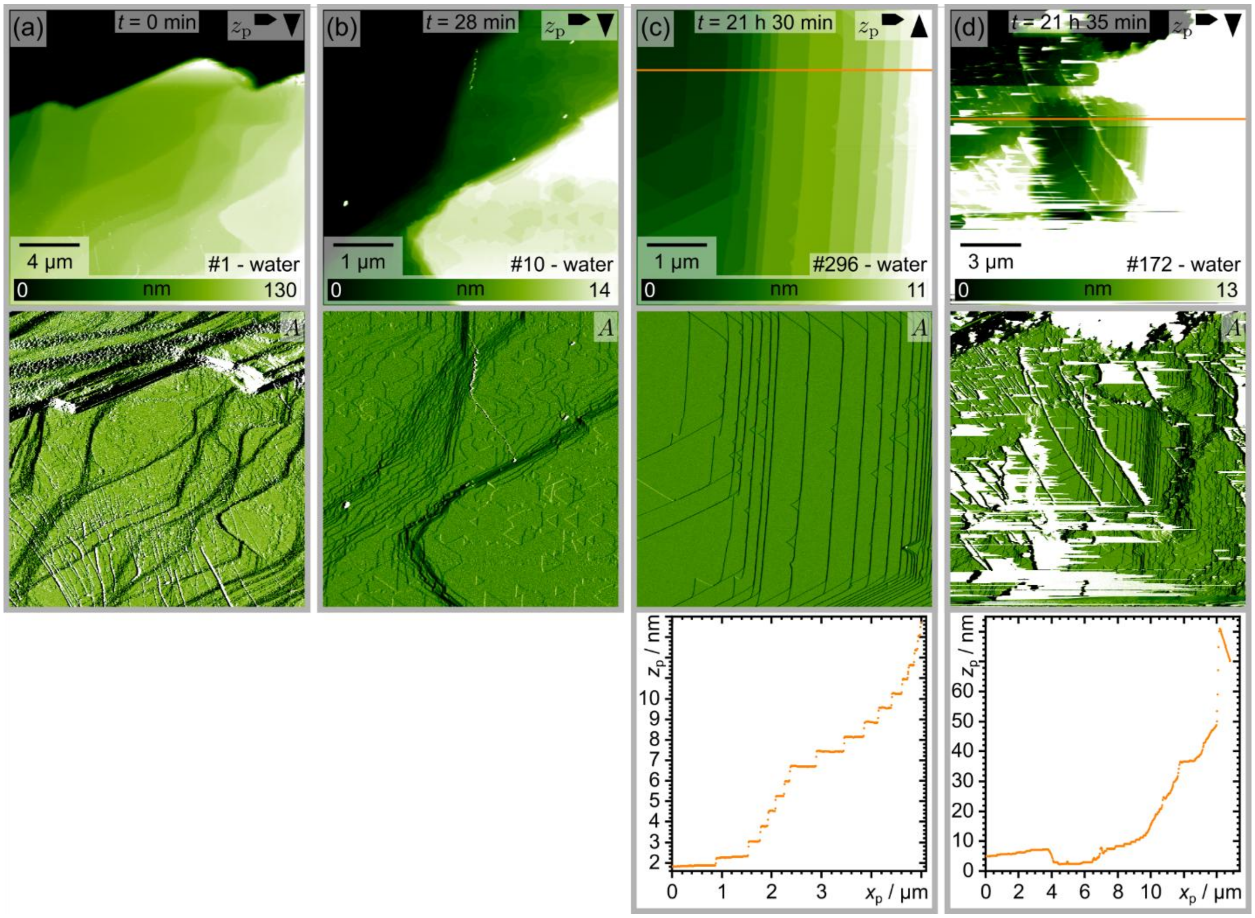

Figure S5. Selected AFM images (height channel z_p and corresponding amplitude A images) from a series taken at the Ag-terminated AgI(0001)-water interface. The height profiles shown in (c) and (d) were extracted along the orange lines. The time t indicates the elapsed time between the images shown in (b) – (d) relative to the image in (a). The image number in the series is shown in the lower right corner, and the arrows in the upper right corner indicate the fast and slow scan directions. A video file "S5_water(0001)Ag.mp4" corresponding to this image series is provided in the Supporting Information.

Section II: Further image series and height profiles of I-terminated (000-1) surfaces cleaved in pure water

Unless otherwise stated in the figure caption, the AFM data was processed as follows. The large-scale AM topography (z_p channel) images were levelled by a mean plane subtraction and fitting a plane through three points. Rows were aligned by a median of differences.

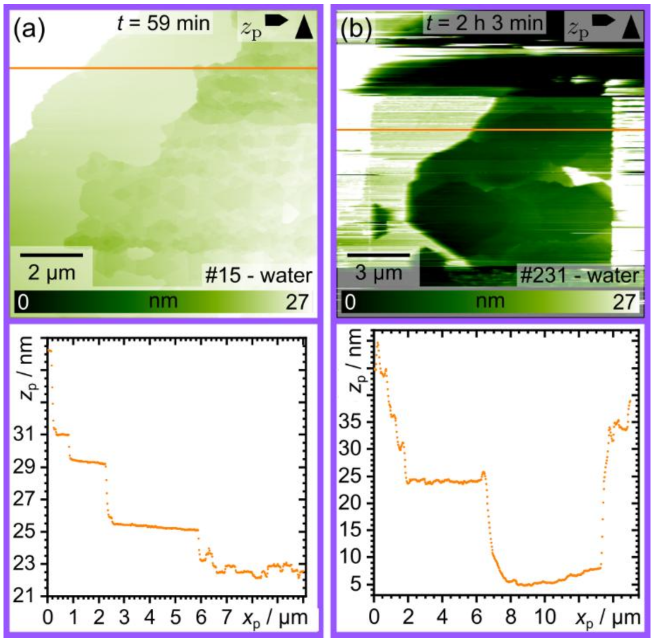

Figure S6. AFM images (height channel z_p) as shown in **Figure 4b** and **4d** in the main text, with height profiles extracted along the orange lines.

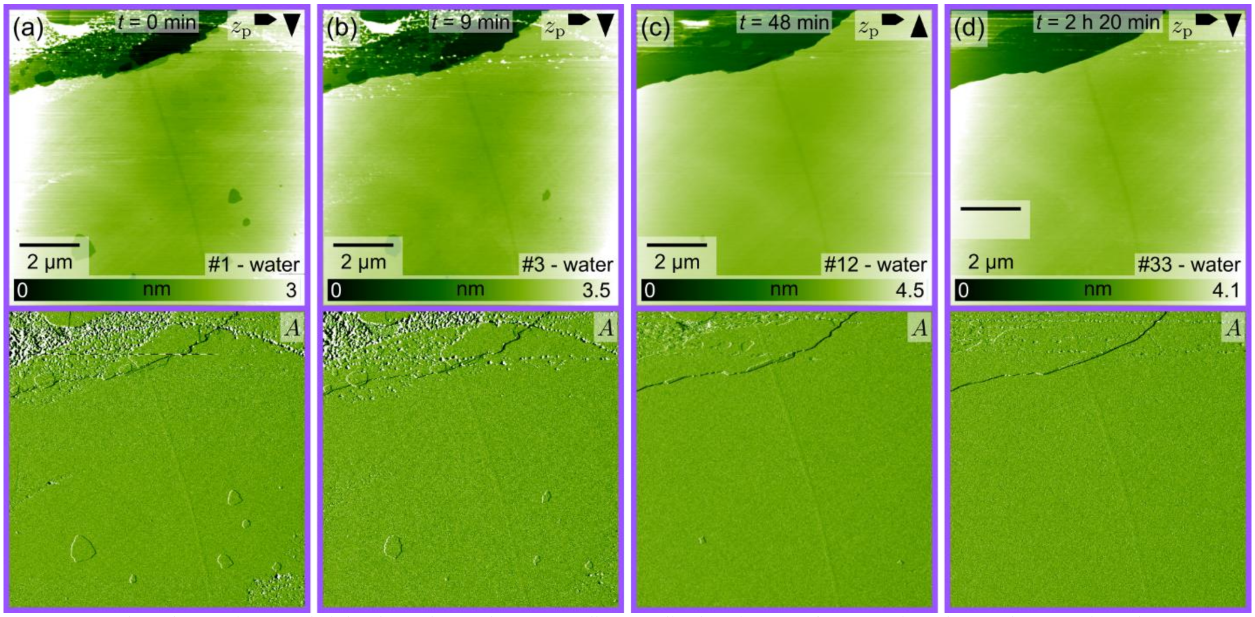

Figure S7. Selected AFM images (height channel z_p and corresponding amplitude A images) from a series taken at the I-terminated AgI(000-1)-water interface. The time t indicates the elapsed time between the images shown in (b) – (d) relative to the image in (a). The image number in the series is shown in the lower right corner, and the arrows in the upper right corner indicate the fast and slow scan directions. A video file "S7_water(000-1)I.mp4" corresponding to this image series is provided in the Supporting Information.

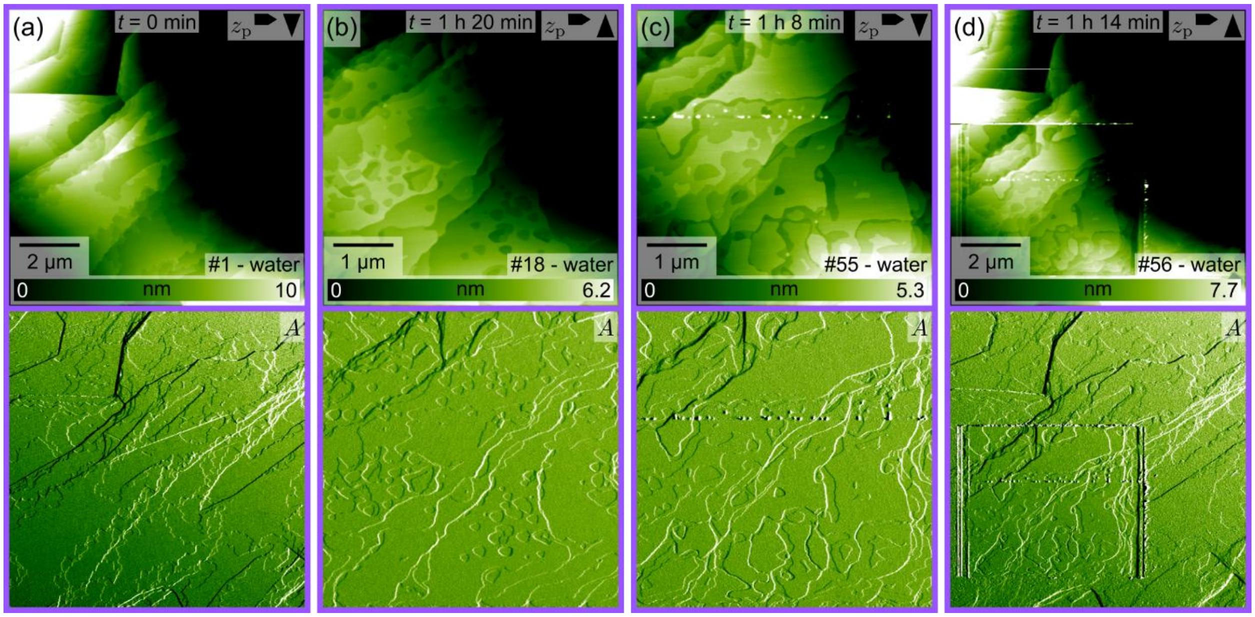

Figure S8. Selected AFM images (height channel z_p and corresponding amplitude A images) from a series taken at the I-terminated AgI(000-1)-water interface. The time t indicates the elapsed time between the images shown in (b) – (d) relative to the image in (a). The image number in the series is shown in the lower right corner, and the arrows in the upper right corner indicate the fast and slow scan directions. A video file "S8_water(000-1)I.mp4" corresponding to this image series is provided in the Supporting Information.

Section III: Image series and height profiles of Ag-terminated (0001) and I-terminated (000-1) surfaces cleaved in 0.1 M and 1 M NaCl aqueous solutions

Unless otherwise stated in the figure caption, the AFM data was processed as follows. The large-scale AM topography (z_p channel) images were levelled by a mean plane subtraction and fitting a plane through three points. Rows were aligned by a median of differences.

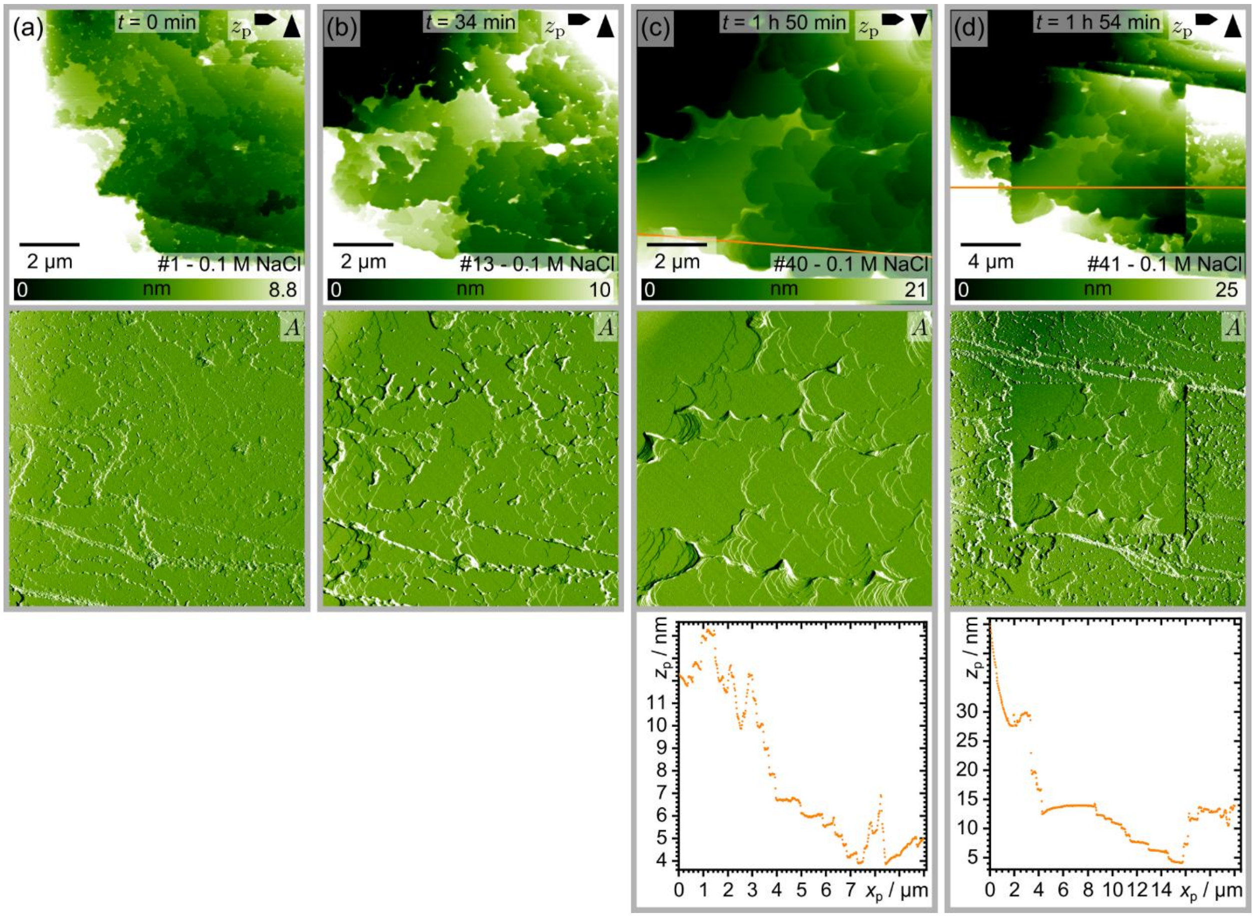

Figure S9. Selected AFM images (height channel z_p and corresponding amplitude A images) from a series taken at the interface between the Ag-terminated AgI(0001) plane and a 0.1 M NaCl aqueous solution. The height profiles shown in (c) and (d) were extracted along the orange lines. The time t indicates the elapsed time between the images shown in (b) – (d) relative to the image in (a). The image number in the series is shown in the lower right corner, and the arrows in the upper right corner indicate the fast and slow scan directions. A video file "S9_01M_NaCl(0001)Ag.mp4" corresponding to this image series is provided in the Supporting Information.

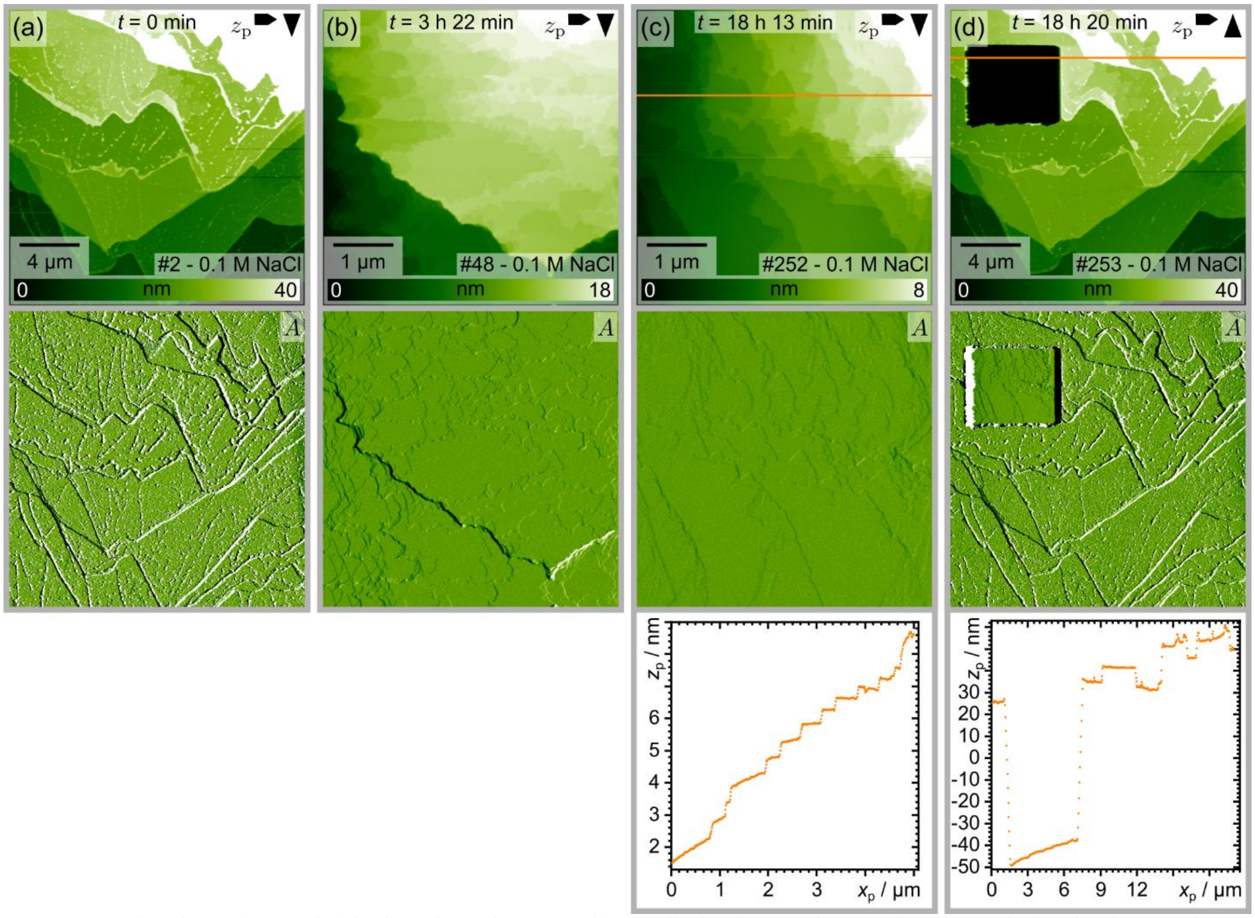

Figure S10. Selected AFM images (height channel z_p and corresponding amplitude A images) from a series taken at the interface between the Ag-terminated AgI(0001) plane and a 0.1 M NaCl aqueous solution. The height profiles shown in (c) and (d) were extracted along the orange lines. As shown in (d), a deep hole is formed during scanning. The pronounced hole formation is tentatively ascribed to the long scanning time of this series rather than the presence of NaCl. The time t indicates the elapsed time between the images shown in (b) – (d) relative to the image in (a). The image number in the series is shown in the lower right corner, and the arrows in the upper right corner indicate the fast and slow scan directions. A video file “S10_01M_NaCl(0001)Ag.mp4” corresponding to this image series is provided in the Supporting Information.

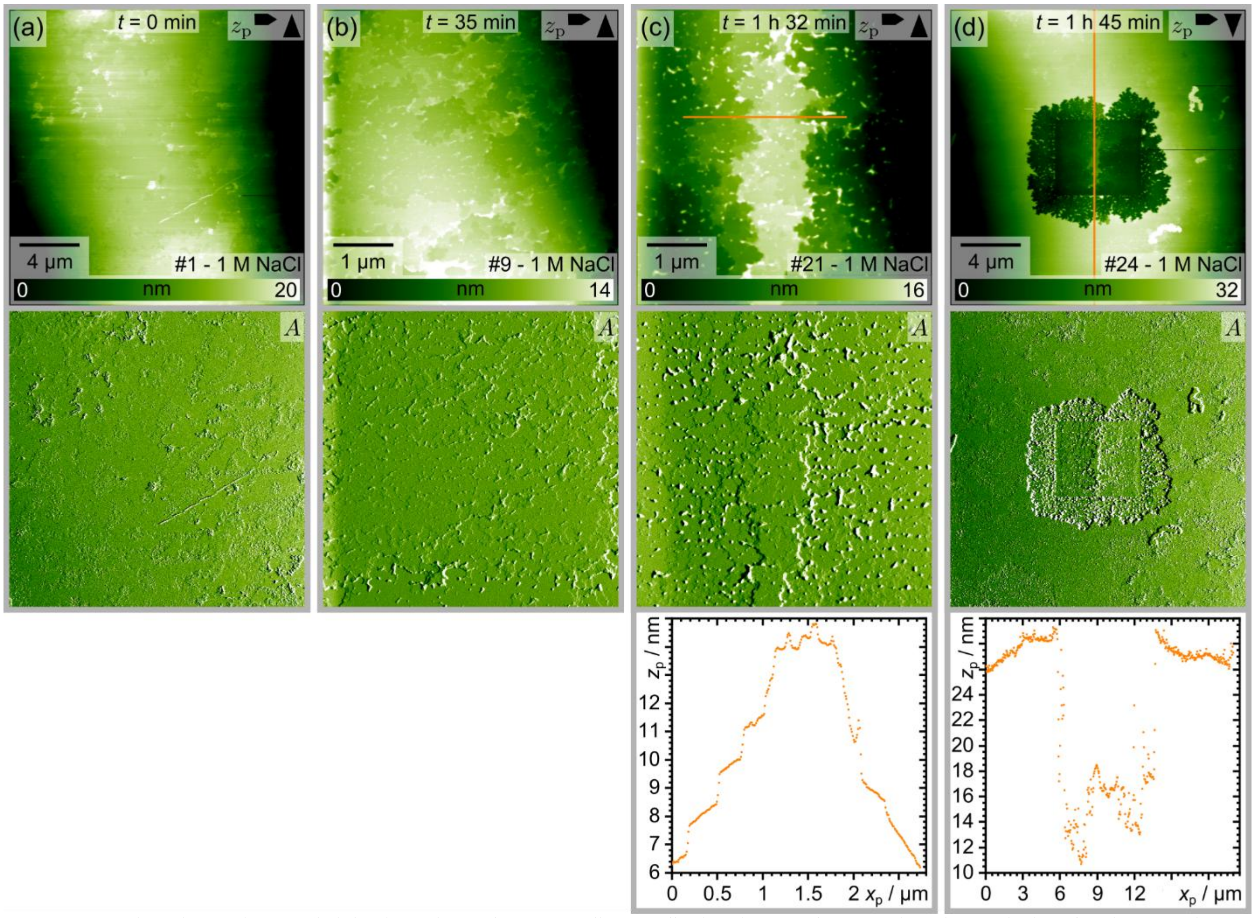

Figure S11. Selected AFM images (height channel z_p and corresponding amplitude A images) from a series taken at the interface between the Ag-terminated AgI(0001) plane and a 1 M NaCl aqueous solution. The height profiles shown in (c) and (d) were extracted along the orange lines. The time t indicates the elapsed time between the images shown in (b) – (d) relative to the image in (a). The image number in the series is shown in the lower right corner, and the arrows in the upper right corner indicate the fast and slow scan directions. A video file "S11_1M_NaCl(0001)Ag.mp4" corresponding to this image series is provided in the Supporting Information.

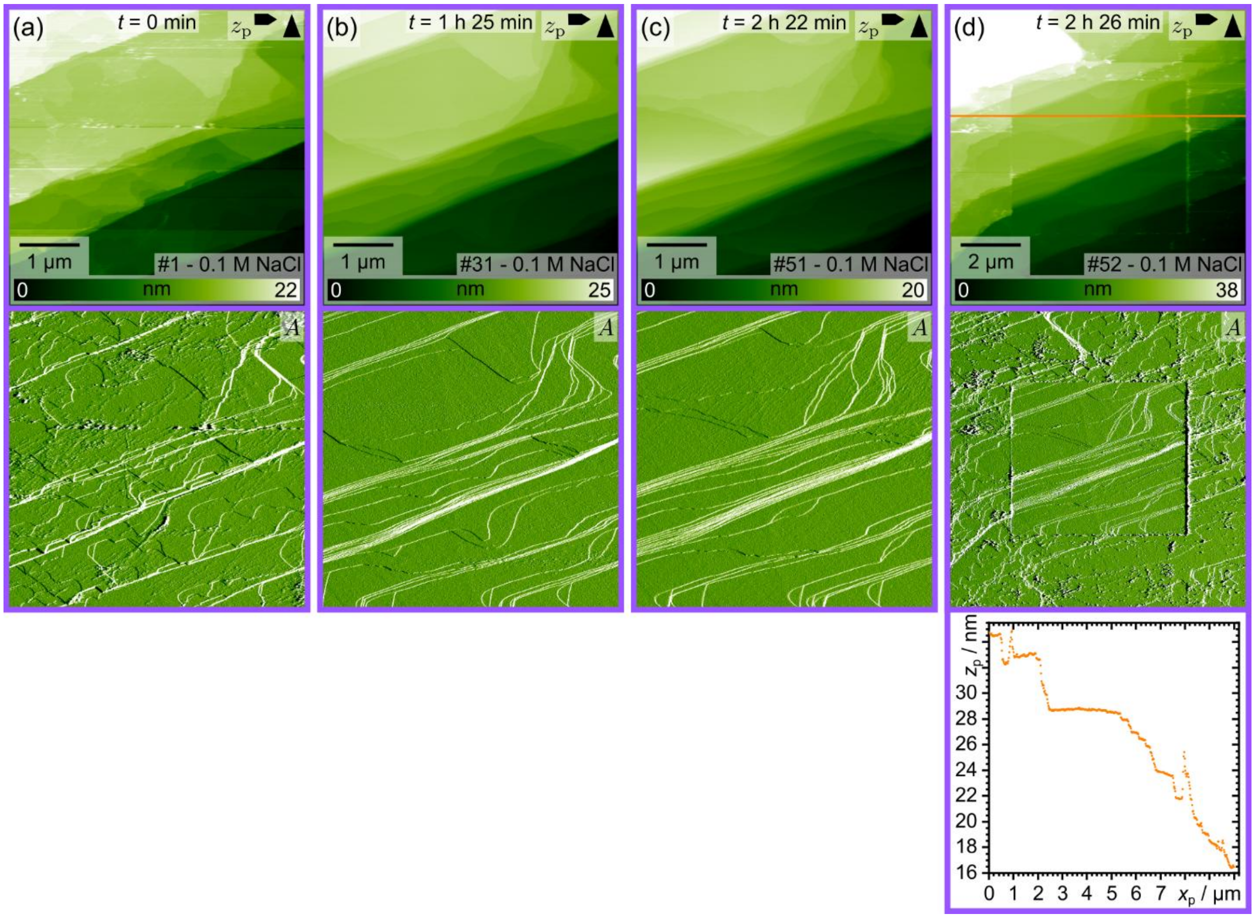

Figure S12. Selected AFM images (height channel z_p and corresponding amplitude A images) from a series taken at the interface between the I-terminated AgI(000-1) plane and a 0.1 M NaCl aqueous solution. The height profile shown in (d) was extracted along the orange line. The time t indicates the elapsed time between the images shown in (b) – (d) relative to the image in (a). The image number in the series is shown in the lower right corner, and the arrows in the upper right corner indicate the fast and slow scan directions. A video file "S12_0.1M_NaCl(000-1)I.mp4" corresponding to this image series is provided in the Supporting Information.

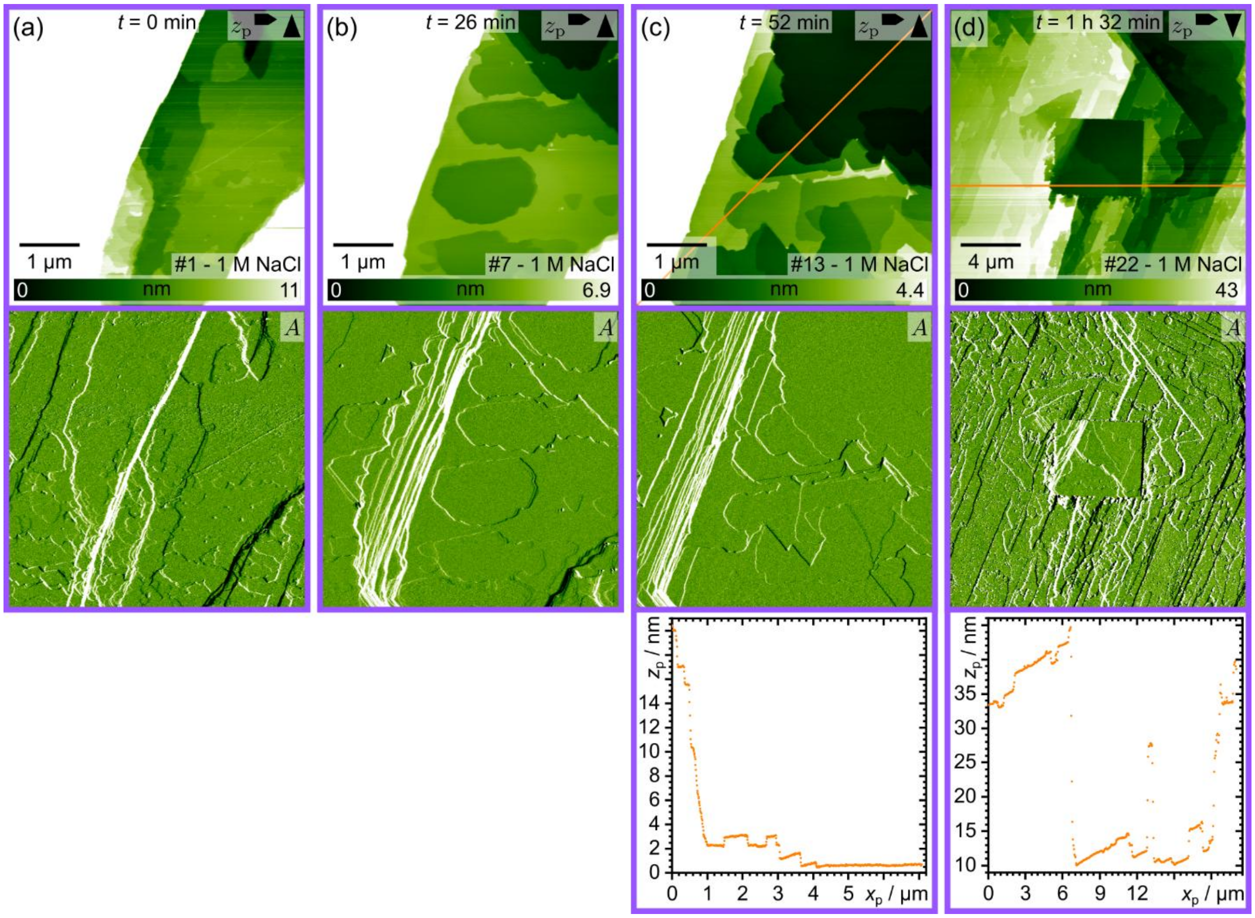

Figure S13. Selected AFM images (height channel z_p and corresponding amplitude A images) from a series taken at the interface between the I-terminated AgI(000-1) plane and a 1 M NaCl aqueous solution. The height profiles shown in (c) and (d) were extracted along the orange lines. The time t indicates the elapsed time between the images shown in (b) – (d) relative to the image in (a). The image number in the series is shown in the lower right corner, and the arrows in the upper right corner indicate the fast and slow scan directions. A video file "S13_1M_NaCl(000-1)I.mp4" corresponding to this image series is provided in the Supporting Information.

Section IV: Confocal microscopy images of Ag-terminated (0001) surfaces

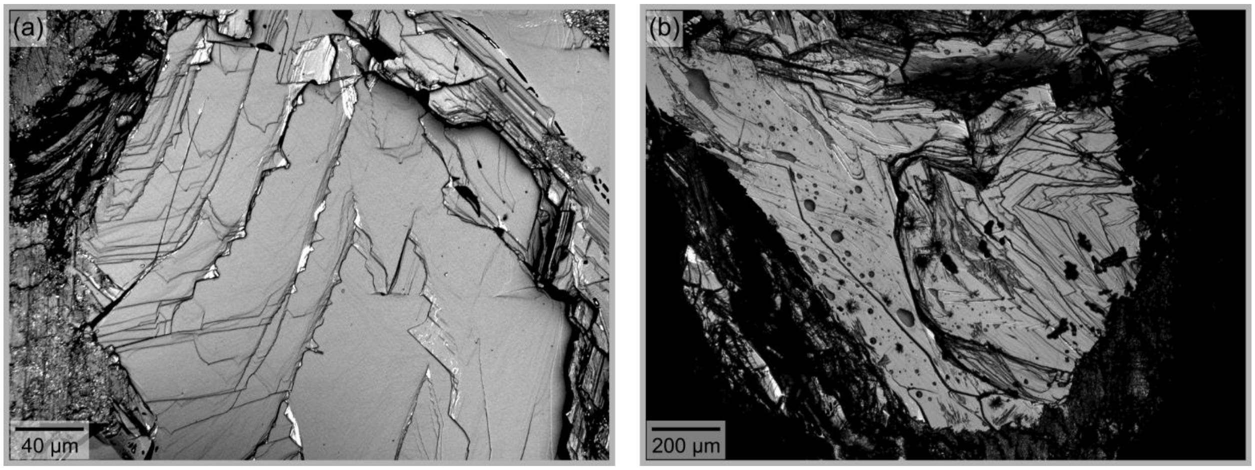

Figure S14. Images of two cleaved AgI samples taken with a DCM8 optical microscope (Leica Microsystems GmbH, Germany) in a confocal mode. a) Favorable AgI(0001)-Ag cleavage plane for AFM experiments, without macroscopically visible impurities. 50-fold magnification. Brightness and contrast were increased by 30 % and 10 %, respectively. b) AgI(0001)-Ag cleavage plane with macroscopically visible droplets and impurities. Ten-fold magnification. Brightness was increased by 10 %.

Section V: Further image series and height profiles of Ag-terminated (0001) and I-terminated (000-1) surfaces cleaved in *n*-dodecane

Unless otherwise stated in the figure caption, the AFM data was processed as follows. The large-scale AM topography (z_p channel) images were levelled by a mean plane subtraction and fitting a plane through three points. Rows were aligned by a median of differences.

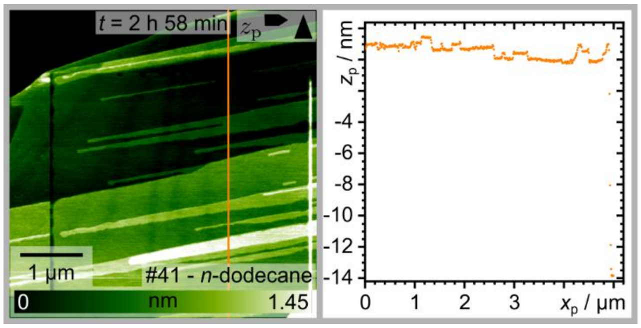

Figure S15. AFM image (height channel z_p) as shown in **Figure 5c** in the main text, with a height profile extracted along the orange line.

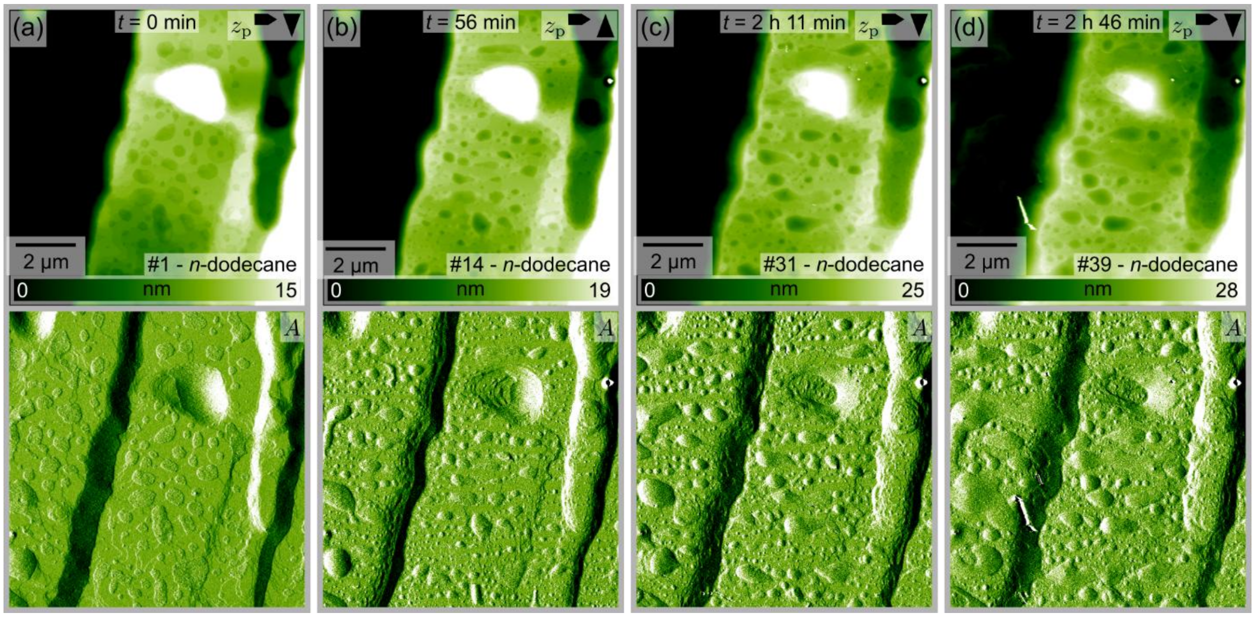

Figure S16. Selected AFM images (height channel z_p and corresponding amplitude A images) from a series taken at the Ag-terminated AgI(0001)-*n*-dodecane interface. The time t indicates the elapsed time between the images shown in (b) – (d) relative to the image in (a). The image number in the series is shown in the lower right corner, and the arrows in the upper right corner indicate the fast and slow scan directions. A video file "S16_dodecane(0001)Ag.mp4" corresponding to this image series is provided in the Supporting Information.

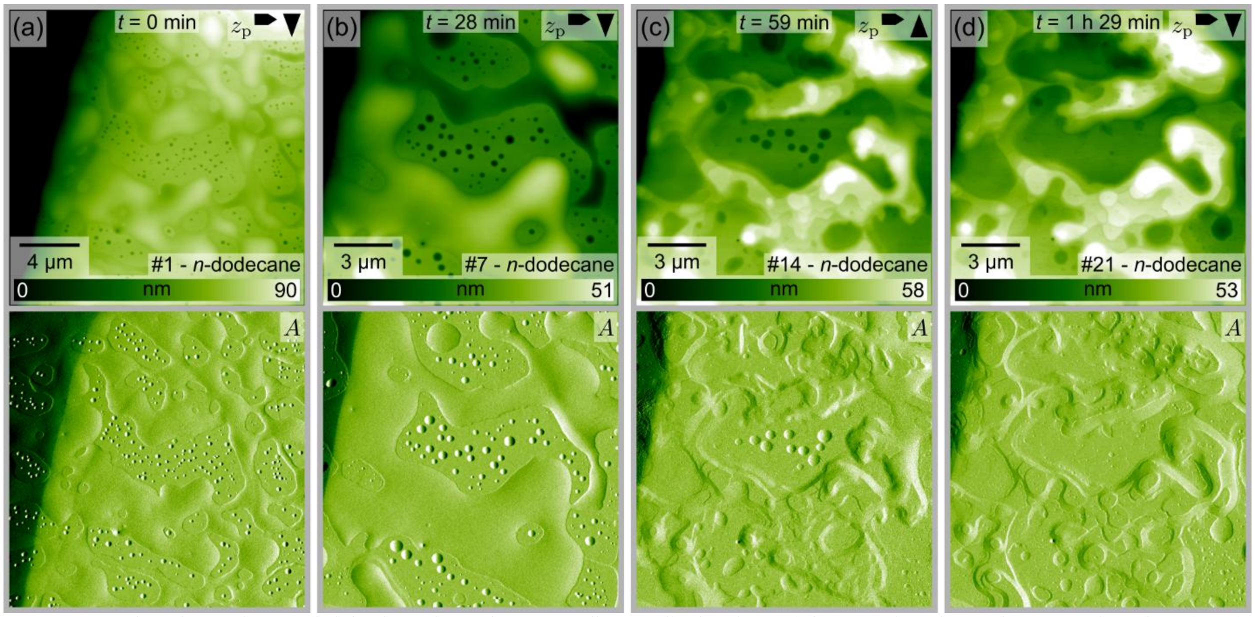

Figure S17. Selected AFM images (height channel z_p and corresponding amplitude A images) from a series taken at the Ag-terminated AgI(0001)-*n*-dodecane interface. The time t indicates the elapsed time between the images shown in (b) – (d) relative to the image in (a). The image number in the series is shown in the lower right corner, and the arrows in the upper right corner indicate the fast and slow scan directions. A video file "S17_dodecane(0001)Ag.mp4" corresponding to this image series is provided in the Supporting Information.

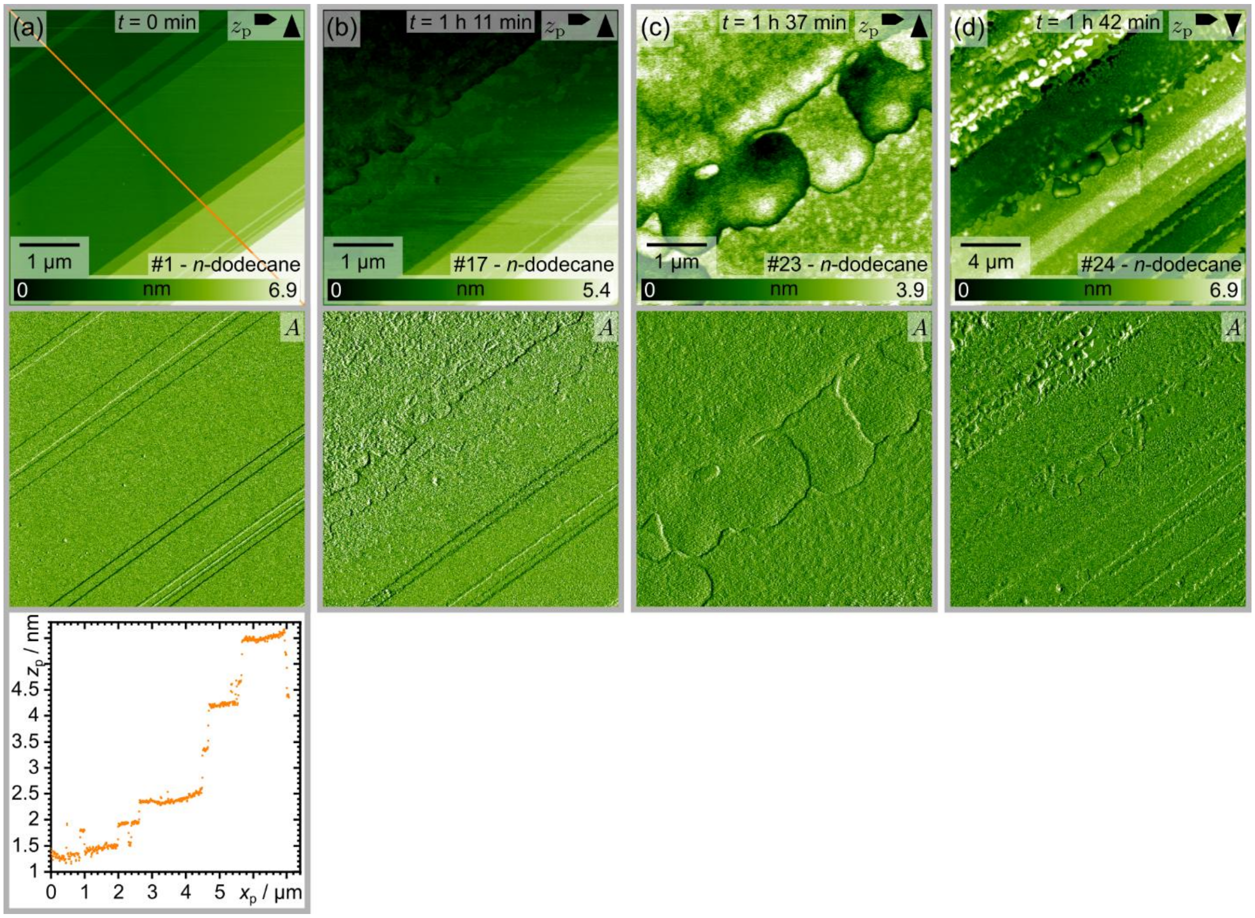

Figure S18. Selected AFM images (height channel z_p and corresponding amplitude A images) from a series taken at the Ag-terminated AgI(0001)-n-dodecane interface. The height profile shown in (a) was extracted along the orange line. The time t indicates the elapsed time between the images shown in (b) – (d) relative to the image in (a). The image number in the series is shown in the lower right corner, and the arrows in the upper right corner indicate the fast and slow scan directions. The z_p-images in (c) and (d) could not be levelled by fitting a plane through three points. A video file "S18_dodecane(0001)Ag.mp4" corresponding to this image series is provided in the Supporting Information.

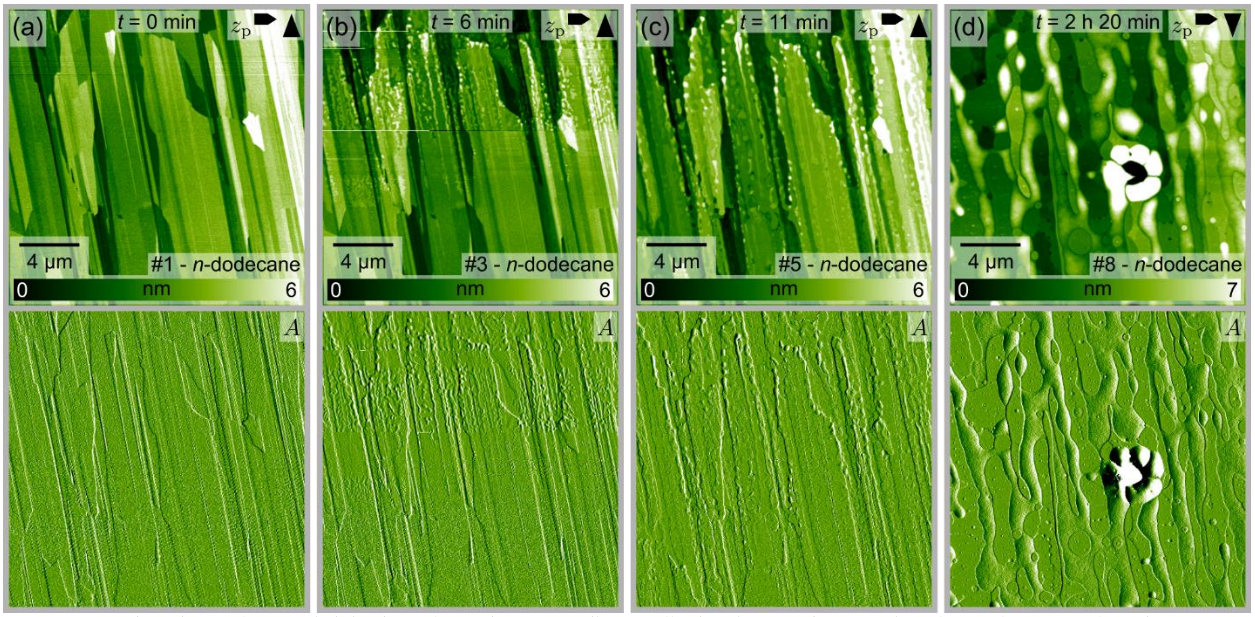

Figure S19. Selected AFM images (height channel z_p and corresponding amplitude A images) from a series taken at the Ag-terminated AgI(0001)-*n*-dodecane interface. Between the measurements of the images in (c) and (d), atomic-scale measurements were performed which led to the distinctive feature visible in (d). The time t indicates the elapsed time between the images shown in (b) – (d) relative to the image in (a). The image number in the series is shown in the lower right corner, and the arrows in the upper right corner indicate the fast and slow scan directions. A video file "S19_dodecane(0001)Ag.mp4" corresponding to this image series is provided in the Supporting Information.

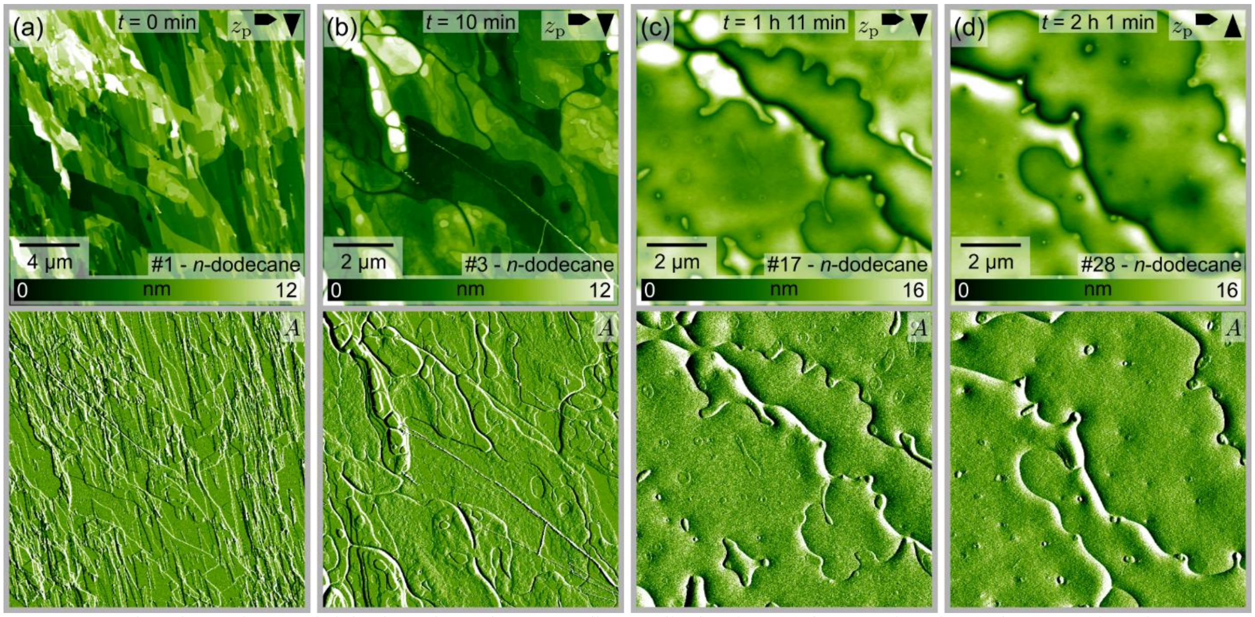

Figure S20. Selected AFM images (height channel z_p and corresponding amplitude A images) from a series taken at the Ag-terminated AgI(0001)-*n*-dodecane interface. The time t indicates the elapsed time between the images shown in (b) – (d) relative to the image in (a). The image number in the series is shown in the lower right corner, and the arrows in the upper right corner indicate the fast and slow scan directions. Only the z_p-image in (a) could be levelled by fitting a plane through three points. A video file "S20_dodecane(0001)Ag.mp4" corresponding to this image series is provided in the Supporting Information.

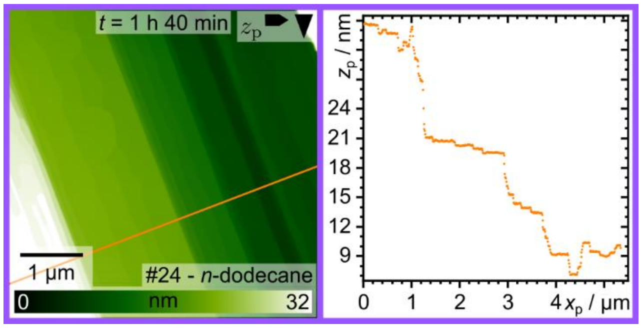

Figure S21. AFM image (height channel z_p) as shown in **Figure 6b** in the main text, with a height profile extracted along the orange line.

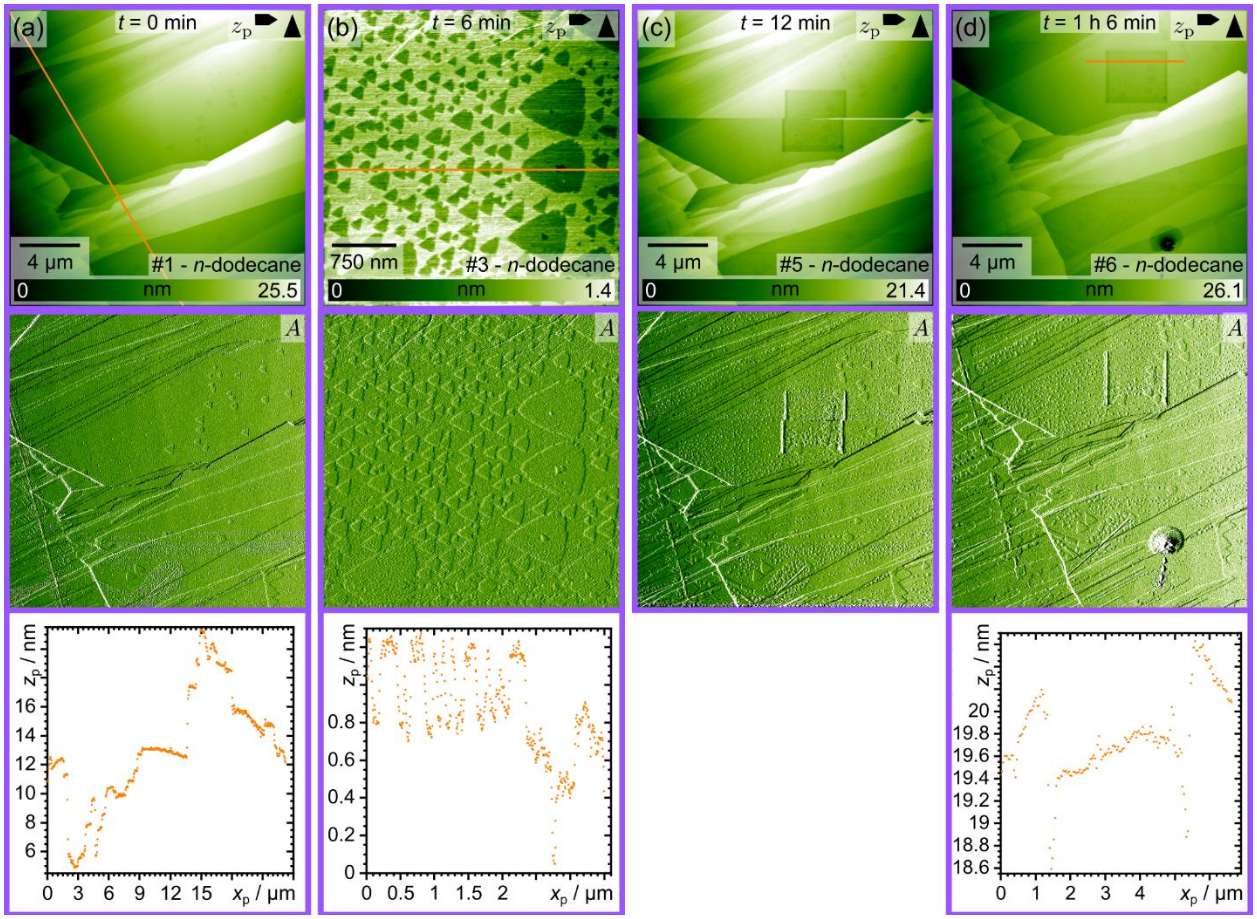

Figure S22. Selected AFM images (height channel z_p and corresponding amplitude A images) from a series taken at the I-terminated AgI(000-1)-*n*-dodecane interface. Compared to the image in (c), the image in (d) has an *xy*-offset. Between the measurements of those two images, atomic-scale measurements were performed which led to the distinctive feature visible in the lower right corner in (d). The height profiles shown in (a), (b) and (d) were extracted along the orange lines. The time t indicates the elapsed time between the images shown in (b) – (d) relative to the image in (a). The image number in the series is shown in the lower right corner, and the arrows in the upper right corner indicate the fast and slow scan directions. A video file "S22_dodecane(000-1)I.mp4" corresponding to this image series is provided in the Supporting Information.

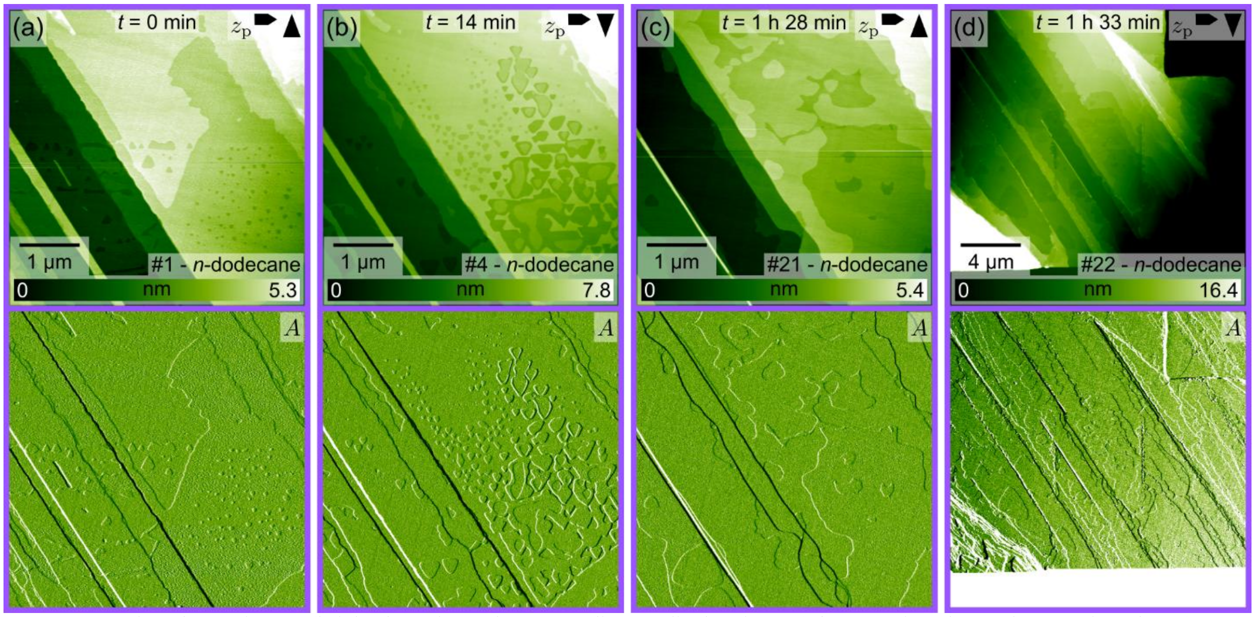

Figure S23. Selected AFM images (height channel z_p and corresponding amplitude A images) from a series taken at the I-terminated AgI(000-1)-*n*-dodecane interface. The time *t* indicates the elapsed time between the images shown in (b) – (d) relative to the image in (a). The image number in the series is shown in the lower right corner, and the arrows in the upper right corner indicate the fast and slow scan directions. A video file "S23_dodecane(000-1)I.mp4" corresponding to this image series is provided in the Supporting Information

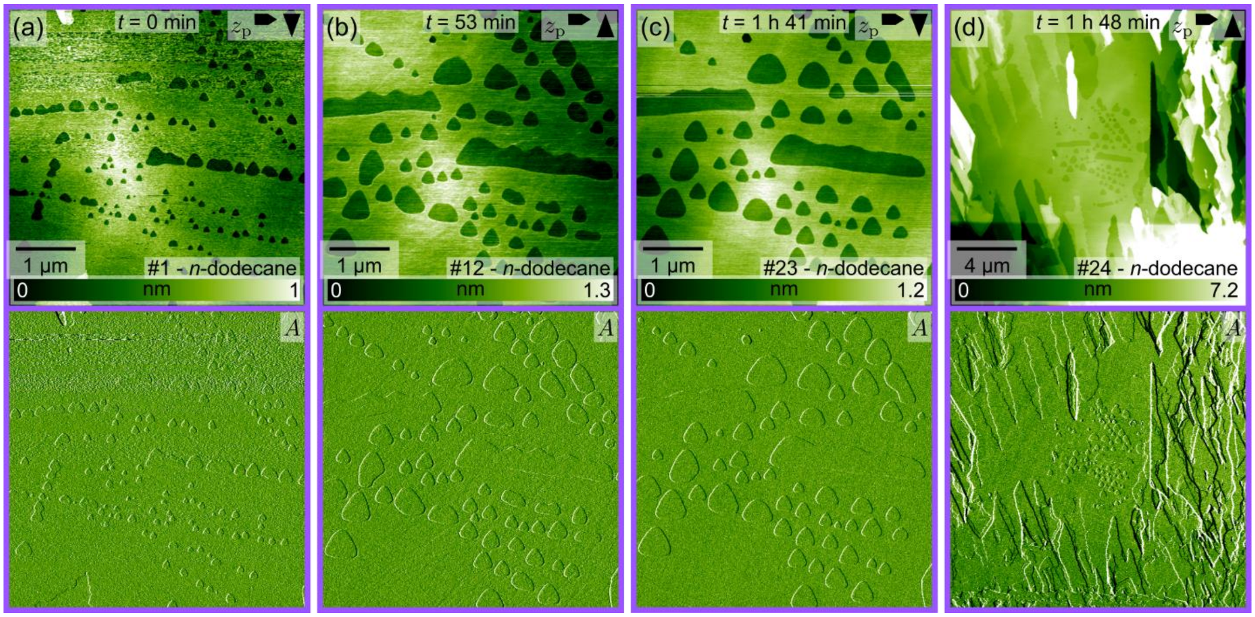

Figure S24. Selected AFM images (height channel z_p and corresponding amplitude A images) from a series taken at the I-terminated AgI(000-1)-*n*-dodecane interface. The time *t* indicates the elapsed time between the images shown in (b) – (d) relative to the image in (a). The image number in the series is shown in the lower right corner, and the arrows in the upper right corner indicate the fast and slow scan directions. A video file "S24_dodecane(000-1)I.mp4" corresponding to this image series is provided in the Supporting Information.

Section VI: Images and height profiles of Ag-terminated (0001) and I-terminated (000-1) surfaces cleaved in 0.1 M and 1 M KI aqueous solutions

Unless otherwise stated in the figure caption, the AFM data was processed as follows. The large-scale AM topography (z_p channel) images were levelled by a mean plane subtraction and fitting a plane through three points. Rows were aligned by a median of differences.

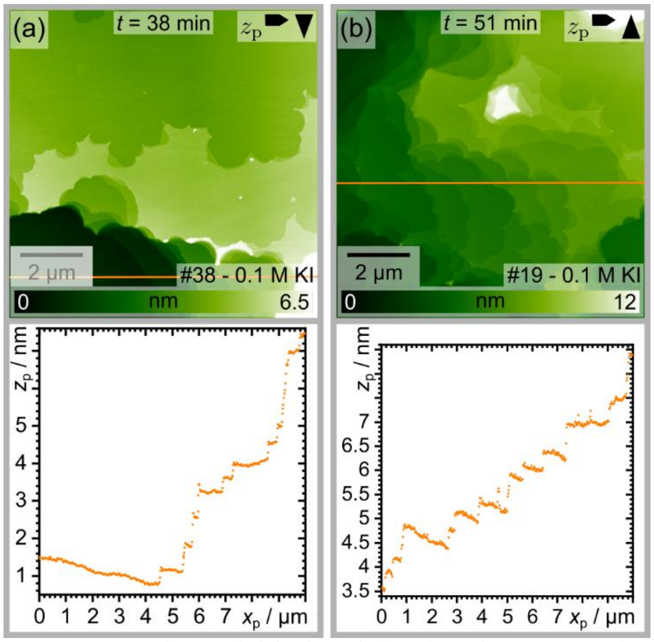

Figure S25. AFM images (height channel z_p) as shown in **Figure 7c** and **7f** in the main text, with height profiles extracted along the orange lines.

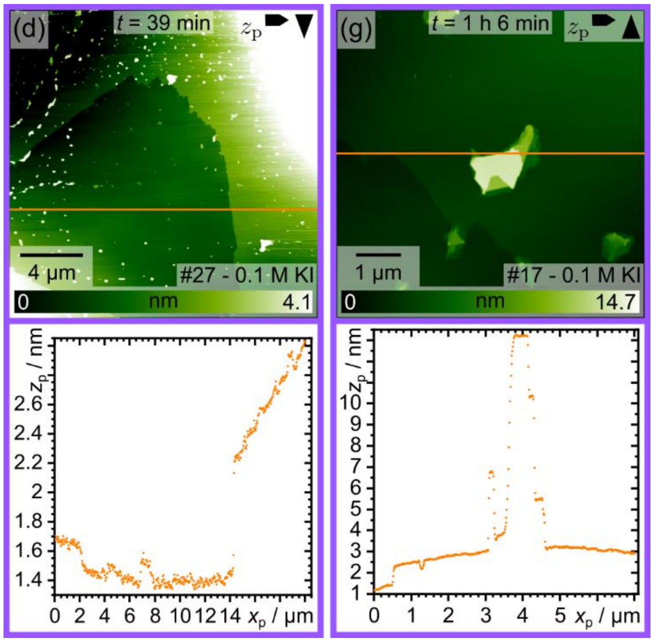

Figure S26. AFM images (height channel z_p) as shown in **Figure 7c** and **7f** in the main text, with height profiles extracted along the orange lines.

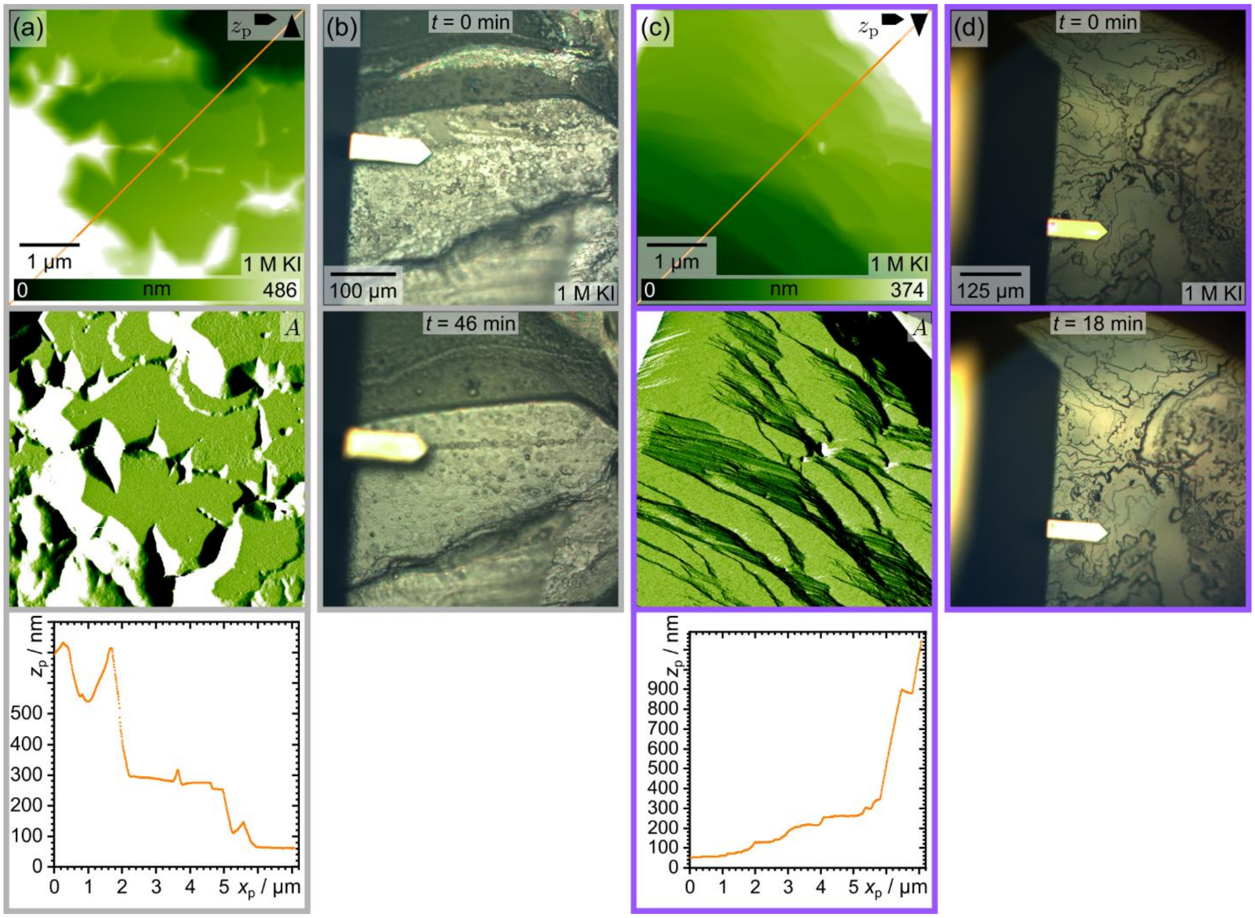

Figure S27. Images of the AgI cleavage planes in a 1 M KI aqueous solution. (a) AFM image (height channel z_p and corresponding amplitude A image) of the Ag-terminated AgI(0001) plane. (b) Images of the Ag-terminated AgI(0001) surface taken with an optical microscope during the AFM experiment. (c) AFM image (height channel z_p and corresponding amplitude A image) of the I-terminated AgI(000-1) plane. (d) Images of the I-terminated AgI(000-1) surface taken with an optical microscope during the AFM experiment. The height profiles shown in (a) and (c) were extracted along the orange lines. The arrows in the upper right corner indicate the fast and slow scan directions and the time t indicates the elapsed time between the lower image relative to the upper image shown in (b) and (d), respectively.

Section VII: Images and height profiles of Ag-terminated (0001) and I-terminated (000-1) surfaces cleaved in 1 M NaI aqueous solutions

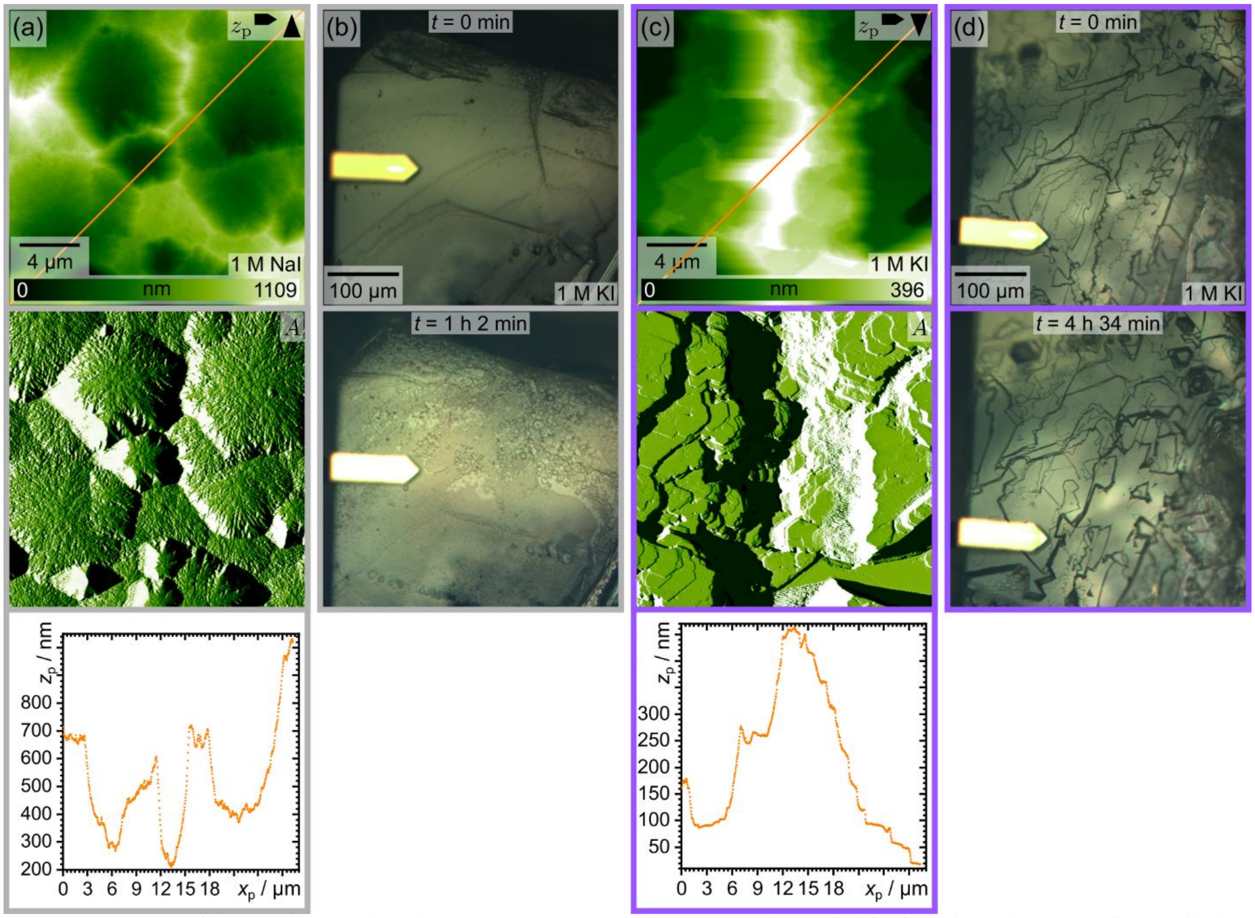

Figure S28. Images of the AgI cleavage planes in a 1 M NaI aqueous solution. (a) AFM image (height channel z_p and corresponding amplitude A image) of the Ag-terminated AgI(0001) plane. (b) Images of the Ag-terminated AgI(0001) surface taken with an optical microscope during the AFM experiment. (c) AFM image (height channel z_p and corresponding amplitude A image) of the I-terminated AgI(000-1) plane. (d) Images of the I-terminated AgI(000-1) surface taken with an optical microscope during the AFM experiment. The height profiles shown in (a) and (c) were extracted along the orange lines. The arrows in the upper right corner indicate the fast and slow scan directions and the time t indicates the elapsed time between the lower image relative to the upper image shown in (b) and (d), respectively.
